# Symptoms of long COVID in children and adolescents: a scoping review

**DOI:** 10.1590/1980-220X-REEUSP-2024-0435en

**Published:** 2025-08-04

**Authors:** Ariel Courbassier Santos de Gusmão, Ana Cristina Ribeiro La Scaléa, Silvia Carla da Silva André Uehara

**Affiliations:** 1 Universidade Federal de São Carlos, Departamento de Enfermagem, São Carlos, SP, Brazil.

**Keywords:** Child, Adolescent, Post-Acute COVID-19 Syndrome, COVID-19, Signs and Symptoms

## Abstract

**Objective:**

To map the symptoms of Long Covid (LC) presented by children and adolescents.

**Method:**

This is a scoping review, using the search engines Web of Science, Scopus, Virtual Health Library, and PUBMED, following the principles of the Joanna Briggs Institute.

**Results:**

Sixteen studies were selected, which showed that fatigue, headache, dyspnea, and cough were the most frequent symptoms of LC. There is a tendency for the development of child-adolescent LC related to the increase in age range, and the correlation between LC and predominant sex proved to be inconclusive. The presence of comorbidities, such as obesity, respiratory, neurological and renal diseases, was the most reported and a study showed an association between Covid-19 vaccine protection and LC.

**Conclusion:**

This review points to a plurality of symptomatic manifestations of LC in children and adolescents, changing according to age group and health history.

## INTRODUCTION

Five years after the start of the pandemic, there has been an endemic spread of Covid-19 disease and a high frequency of Long Covid (LC) symptoms in the population, including children and adolescents^(1)^. LC or post-Covid condition are terms adopted to describe persistent symptoms, acquired after previous infection by Covid-19, and characterized by the presence of symptoms after three months from their onset and by their continuity for at least two months, and by not being clarified by other tests and illnesses^(2)^.

From this perspective, around 10 to 20% of people in the general population who have been infected with Covid-19 may experience prolonged symptoms. Although the specific groups most likely to develop LC are not yet clear, there is evidence that women and patients who have had the severe form of Covid-19 are more susceptible^(3)^. Thus, of the more than 651 million reported cases of Covid-19, as well as many undocumented ones, it is estimated that at least 65 million people developed LC, with 50 to 70% of cases having a previous history of hospitalization, 10 to 30% without hospitalization, and 10 to 12% with vaccination^(4,5,6,7)^.

Biologically, LC may be related to an aggregated immune or inflammatory response and the causes may be associated with the existence of an imbalance in the intestinal flora^(8)^, oxidative damage, and decreased antioxidant protections^(9)^, signs of malfunctioning metabolic activity^(10)^_,_ and immune defense mechanisms, endothelial cell damage and SARS-CoV-2 latency; however, the pathogenesis is not completely understood^(11)^.

In this context, it was observed that the symptoms of LC among children and adolescents can be distinct and the respiratory system is the most affected, frequently presenting cough, shortness of breath, and chest pain. However, reports of symptoms related to gastrointestinal disorders, diarrhea, nausea, difficulty concentrating, and irritability may also be observed^(1,12)^.

Thus, although LC in children and adolescents can compromise their quality of life, negatively interfering in daily, social, and school activities and even affecting both physical and cognitive development, there is a lack of literature on this topic in this population. The symptoms of LC may be different from those seen in adults or older people and may go unnoticed, impacting the quality of life of children and hindering diagnosis and adequate treatment^(1,12)^. Thus, aiming at searching for persistent gaps in the literature regarding symptoms in the child and adolescent population, this study proposes to map the symptoms of LC presented by children and adolescents.

## METHOD

### Design of Study

This is a scoping review based on the principles advocated by the Joanna Briggs Institute (JBI). The research followed these steps: (1) identification of the research question, (2) identification of relevant studies, (3) selection of studies, (4) data extraction, (5) grouping, summary, and reporting of results, (6) consultation, (7) analysis of evidence, (8) presentation of data and results, and (9) summary of results and evidence, elaboration of conclusions^(13)^.

### Defining The Research Question

To conduct the search in the review, the definition of the research question was carried out using the acronym “PCC” (population, concept, and context), with “P” being children and adolescents, “C” symptoms of long covid, and “C”, post pandemic: What are the symptoms of LC presented by children and adolescents, considering age, sex, comorbidities, and vaccination status in the post-pandemic period?

### Search Strategy and Inclusion and Exclusion Criteria

The bibliographic research was conducted in the electronic databases: *Web of Science*, *US National Library of Medicine National Institutes of Health* PUBMED – NCB, Scopus and Virtual Health Library (VHL), using descriptors and synonyms referenced in the Health Sciences Descriptor (DeCS) and in *Medical Subject Headings* (MeSH): *criança; adolescente; covid longa; Sintomas; Síndrome Pós-covid*, in Portuguese; and, in English: Child, Adolescent, Long COVID, Post-Acute COVID-19 Syndrome, Symptoms (Chart 1).

**Chart 1:** Search strategies used in databases – São Carlos, SP, Brazil, 2024.

Database	Strategy
VIRTUAL HEALTH LIBRARY	(child) AND (“Long Covid”) AND (symptoms)
WEB OF SCIENCE	((((AB = (Child)) OR AB = (“Adolescent”)) AND TI = (Long COVID)) OR TI = (“Post-Acute COVID-19 Syndrome”)) AND AB = (“Symptoms”)
SCOPUS	(ABS (“Child”) OR ABS (“Adolescent”) AND ABS (“Long COVID”) OR ABS (“Post-Acute COVID-19 Syndrome”) AND ABS (“Symptoms”))
PUBMED	(((“Child”[Abstract] OR “Adolescent”[Abstract]) AND “Long Covid”[Abstract]) OR “Post-Acute COVID-19 Syndrome”[Abstract]) AND “Symptoms” [Abstract]

### Extraction and Selection of Studies

The inclusion criteria were defined as original articles, published in English, Portuguese, or Spanish, regardless of the year of publication, indexed in one of the previously cited databases. Exclusion criteria were defined as duplicate articles, studies that were not available in full, and studies whose titles and abstracts did not fall within the scope of the investigation. In addition, the reference catalogues of the studies were also consulted.

After this step, the references were exported to the web application StArt (*State of the Art through Systematic Review*), for the selection of studies at two levels. The first selection was made by reading titles and abstracts, followed by reading the full article. Eligible studies were retrieved in full and evaluated by two researchers individually. In both stages, differences were debated until an agreement was reached.

### Preparation of The Review

The PRISMA-Sr guidelines were used to select the articles (*extension for scoping reviews),* to collect the following information: author, year, and location of the study, objective, study design, objective and main results. In response to the guiding question of this study, the results found were organized into tables and descriptive texts^(14)^.

## RESULTS

**Figure 1:**
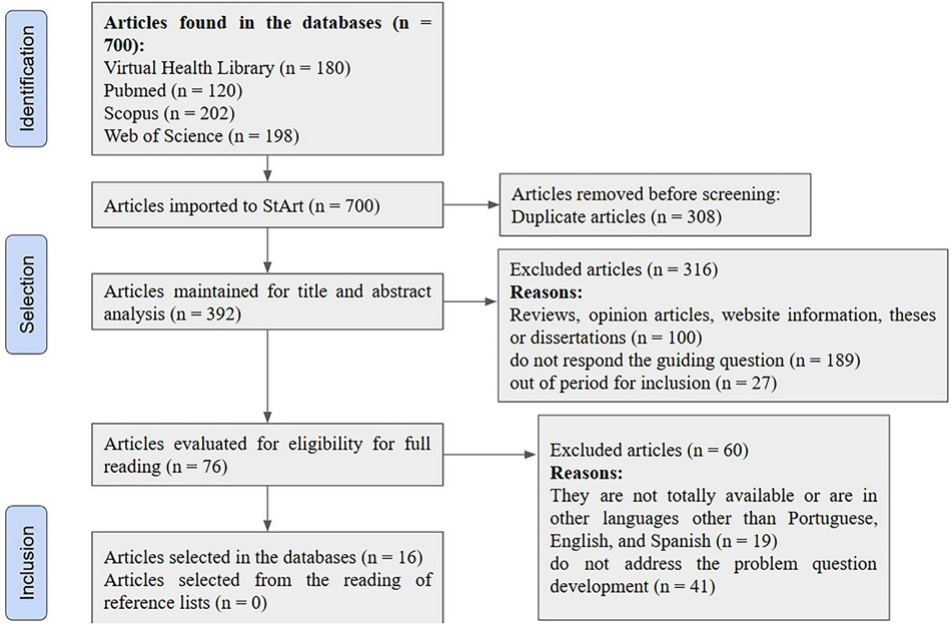
Flowchart and application of study selection according to PRISMA-PCR guidelines. São Carlos, SP, Brazil, 2025.

A total of 700 articles were found in the databases, 308 of which were excluded because they were duplicates. When analyzing the 392 selected, 316 were excluded because they were reviews and opinion articles. Thus, 76 studies were analyzed, of which 60 were excluded because they did not answer the problem question, with 16 articles, published between 2021 and March 2024, being selected (Figure 1).

It should be noted that all studies were published in English and, regarding the type of study, 7 (43.8%) were cohort studies, 4 (25%) case-control studies, 3 (18.8%) cross-sectional studies, 1 (6.2%) case study, and 1 (6.2%) case series (Chart 2).

**Chart 2:** Description of selected articles, according to author, year, location, title, objective, type of study and main results – São Carlos, SP, Brazil, 2025.

Author, Year and Place	Objective	Design of Study	Main Results	Main Symptoms
Asadi-Pooya et al., 2021, Iran^(15)^.	To recognize in pediatric patients diagnosed with Long Covid: the occurrence rate, clinical picture, and risk variables.	Cross-sectional study	26 children in the sample showed symptoms of LC, 15 females and 11 males. Comorbidities and vaccination data for this group were not mentioned. The occurrence rate of LC was more than 40% in hospitalized patients during the acute phase of Covid-19.	Asthenia, exhaustion, inability to exercise, presence of excessive secretion in the airways, cough, arthralgia, headache, sleep disorders, and myalgia.
Ashkenazi-Hoffnung et al., 2021, Israel^(16)^.	To analyze the clinical picture of 90 individuals from the youth population who presented symptoms of Long Covid.	Cohort study	The sample proportion in relation to the sex of 90 children was 1.4 male/1 female. Furthermore, the most frequent comorbidities were attention deficit hyperactivity disorder, immunodeficiency, autoimmune or inflammatory disease, asthma, and anxiety disorder or depression. Vaccination data were not evaluated. Most frequent symptoms of LC were Moreover, a 58.9% reduction in daily performance from usual routines was observed.	Exhaustion, difficulty breathing, and muscle pain, especially in the 11 to 18 age group. Furthermore, tinnitus, very repetitive behaviors, increased heart rate, cough, tremors, joint pain, weight loss of more than 5% of the initial weight, clinical manifestations of the digestive system, taste and smell disorders, alopecia, headache, paresthesia, and angina; in addition to concentration deficit, memory deficit, and sleep disorders.
Roge et al., 2021, Latvia^(17)^.	To recognize and investigate, in the children’s group, the multiple effects of a long infection by the SARS-COV-2 virus, in addition to exposing the contrasts between the continuous symptoms of Covid and other infectious pathologies.	Case control study	In the sample classified as having LC symptoms, 105 were female and 131 were male. Symptoms varied with the children’s age, with airway manifestations being more common in young children and babies. Regarding comorbidities, the following stand out: bronchial asthma, gastrointestinal disorders, psychiatric disorders, epilepsy, cystic fibrosis, allergies, atopic dermatitis, congenital anomalies, neurological and urological disorders.	Exhaustion, headache, runny nose, cough, pyrexia, hyposmia/hypogeusia, night sweats, loss of appetite, weight loss, dizziness, arthralgia, dyspnea at rest, dyspnea on physical exertion, muscle pain, cardiac rhythm symptoms, odynophagia, diarrhea, emesis and nausea, increased respiratory rate, alopecia, lymphadenopathy, presence of pulmonary wheezing, aphasia, and dysphagia. Furthermore, concentration deficit, memory deficit, attention deficit, mood swings, stress, and symptoms of anxiety/depression were reported.
Bossley et al., 2022, United Kingdom^(18)^.	To analyze the child’s clinical picture regarding the development of symptoms similar to those of Long Covid.	Cohort study	Eighty-eight children participated in the study, 61% of whom were male. Of the group with prolonged symptoms, two were male. One participant had no underlying diseases and the other had gastroesophageal reflux, Ehlers-Danlos syndrome, genetic alteration of chromosome 6, and immunoglobulin-A insufficiency. Vaccination data were not collected. For 5.5% of participants, LC symptoms were classified as severe and 7% serious, 12.5% were average. Additionally, the severity of acute Covid symptoms and the development of LC were not correlated.	Among these cases, the most common symptom was non-productive cough.
Kuczborska et al., 2022, Poland^(19)^.	To contrast the disparities in symptomatic manifestations of long Covid in immunologically deficient and non-immunologically deficient infants, in addition to assessing the occurrence rate.	Case control study	The age of the participants ranged from 4 months to 17 years, with 37 males and 33 females in the group with immunological deficiency and 41 males and 36 females in the group with immunological normality. The most frequent comorbidities were immunodeficiency, kidney or liver transplants, colitis with ulcers, demyelinating diseases and tumors. In the group without immune deficiency, allergic diseases were the most cited. Regarding vaccination data, 3 immunodeficient children and 20 immunocompetent children were vaccinated against Covid-19, but no further analysis was performed.	The group with normal immunological status presented exhaustion, reduced capacity for physical effort, concentration deficit, and sleep disturbances. Children and adolescents with immunological vulnerability showed a higher frequency of gastroenterological LC symptoms.
Baptista de Lima et al., 2023^(20)^.	To detail the persistent clinical manifestations in long Covid and their potential risks.	Cohort study	Children and adolescents aged 0 to 18 years participated in the study, 54.4% of whom were male. There was an increase in the probability of developing LC by up to 3 times in 12 weeks when there was some comorbidity involved; however, the only one mentioned was obesity. Fatigue may be related to the presence of diseases such as obesity and serious underlying diseases. Vaccination data were not collected.	Pyrexia, cough, runny nose, exhaustion, sleep disorders and fatigue.
Basra et al., 2023, Indonesia^(21)^.	To understand the symptomatic panorama of Long Covid in children.	Cross-sectional study	A total of 214 children and adolescents up to 18 years old were analyzed. Of the 121 participants who had persistent Covid symptoms, 70 were female and 51 were male. Vaccination data and health history were not analyzed.	Exhaustion after physical or intellectual exercise, increased/decreased heart rate, hypotension, imbalance in the body’s thermoregulatory mechanism, loss of appetite, diarrhea, gastralgia, loss of ability to concentrate on new information and respiratory disorders
Bilu et al., 2023, Israel^(22)^.	To analyze in the long term (up to 24 months) the psychological health of young people who obtained a positive and negative PCR test for the SARS-CoV-2 virus and identify whether or not the psychological symptoms are associated with the infection.	Cohort study	22,354 adolescents aged 12 to 17 were analyzed from March 2020 to March 2021. PCR-negative participants had a higher rate of prescription of psychotropic drugs and mental diagnoses (irritability, anxiety and depression). When compared by sex, the results were similar, with a focus on male participants with negative PCR who had a higher incidence of recognition of eating disorders. In this study, it was found that mental symptoms were associated with the effects of the Covid-19 health emergency and not necessarily with infection by the SARS-CoV-2 virus, even in the long term. Vaccination status and comorbidities were not analyzed.	Irritability, anxiety, and depression.
Horikoshi et al., 2023, Japan^(23)^.	To analyze the clinical manifestations and tests of pediatric patients with long Covid.	Case series	24 children and adolescents aged 15 or under participated. Autism, ADHD, developmental disorder, asthma, orthostatic dysautonomia, and hearing deficit were underlying diseases found in the sample. Additionally, 9 participants received 2 doses of the Covid-19 vaccine.	Numbness, headache, taste and smell dysfunction, complete loss of taste, alopecia, extramembranous pain, asthenia, nausea and/or vomiting and sleep dysfunction, extramembranous pain, mental confusion, intramembranous pain, cough, breathing difficulties, diarrhea, tingling, and psychotic symptoms.
Mancino et al., 2023, Italy^(24)^.	To analyze childhood symptoms of Long Covid, incidences, and the appearance of risk indicators.	Cohort study	Of the 697 children and adolescents selected for monitoring, 12.7% were between 0 and 4 years old, 19.3% between 5 and 10 years old; 38.7% between 11 and 15 years old, and 45% between 16 and 18 years old, 48% were male and the comorbidities mentioned were bronchiolitis and previous wheezing, overweight, and obesity. It should be noted that 81 (11.6%) children presented persistent symptoms after 90 days. Vaccination data were not analyzed.	Weakness, nausea, gastrointestinal symptoms, respiratory difficulties, respiratory difficulty with exertion, cough, cognitive, olfactory and taste dysfunctions, headache, weight loss, gastralgia, and concentration deficit
Sakurada et al., 2023, Japan^(25)^.	To use the comparison of LC symptoms in adults and children/adolescents to elucidate school absenteeism rates and the impacts caused by viral changes.	Case control study	A total of 452 cases of LC were analyzed, 398 adults (19 years or older) and 54 adolescents aged 11 to 18 years. Of the adolescents with LC, 31 were female and 23 were male. Only student patients had their vaccination data analyzed: of the 22 students, 11 received 1 dose of the Covid-19 vaccine, 10 received two doses, and only 1 received three doses. It is highlighted that 56% (28) of adolescents stopped attending school due to LC symptoms. In addition, comorbidities were not analyzed.	Severe fatigue, headaches, sleep, smell and taste disorders.
Saniasiaya, 2023, Malaysia^(26)^.	To analyze the pediatric clinical profiles of vertiginous migraine subsequent to the disease caused by Covid-19.	Case study	The first case involved a child who had attacks of vertigo and nausea three to four times a week, 3 months after infection with SARS-CoV-2 and with no previous associated medical history. The second case was of an adolescent with daily complaints of cephalalgia and postural instability, 6 months after SARS-CoV-2 infection and no other associated medical history. The medical interventions proposed for case number 1 were to adopt a balanced lifestyle and practice athletic activities associated with the prescription of vitamin supplements. In his re-evaluation, his clinical condition showed improvements. The medical interventions proposed for case number 2 included a balanced lifestyle, but a medication prescription of a daily tablet of flunarizine 5 mg was implemented.	Vestibular migraine.
Seery et al., 2023, Argentina^(27)^.	To analyze the long-lasting clinical manifestations and risk variables (margin of approximately ninety days) brought about by the disease caused by SARS-CoV-2.	Case control study	A total of 639 cases of Covid-19 were analyzed, with 219 caregivers reporting symptoms of LC in children and an average age of 7 years. The prolongation of symptoms for more than three months was related to the presence of underlying diseases and dysfunctions such as diabetes, kidney and respiratory tract diseases, in addition to the SARS-CoV-2 infection being symptomatic and being in an older age group. Vaccination data were not analyzed.	Weight loss, myalgia, exhaustion, tachycardia, difficulty breathing, cough, constipation or diarrhea, vomiting or nausea, seizures and loss of appetite, concentration and memory deficits, dysgeusia, anosmia, dizziness, headache, depressive and anxiety symptoms, and mood swings.
Calcaterra et al., 2024, Italy^(28)^.	To monitor the clinical history of pediatric patients undergoing hospital care for acute SARS-CoV-2 infection to check for the development of Long Covid.	Cohort study	167 patients participated in the study: 55% were under two years of age, 30% were between 2 and 10 years of age and 14.3% were over 10 years of age, 46.1% were female and 53.9% were male. However, the authors conclude that there is not necessarily a higher prevalence in a particular sex. The most common comorbidities were neurological conditions, gastrointestinal diseases, genetic conditions, diabetes, kidney disease, heart malformations, obesity, chronic malnutrition, asthma, autoimmune diseases, tumors and others. Vaccination data were not analyzed.	Weight loss, insomnia, pulmonary wheezing, gastralgia, fatigue, persistent cough, lack of appetite, myalgia, headache, arthralgia, and mood swings.
Meiere et al., 2024, Latvia^(29)^.	To analyze childhood age groups diagnosed with Long Covid at least 30 days after primary SARS-CoV-2 infection.	Cross-sectional study	The sample consisted of 220 participants under 18 years of age, 56.8% were female and 43.2% were male. Vaccination data and comorbidities were not analyzed.	Epigastralgia, extreme fatigue, headache, mood swings, restricted physical capacity, drowsiness, productive cough, rhinorrhea, memory deficit, tachycardia and angina.
Razzaghi et al., 2024, United States^(30)^.	To measure the results of vaccine protection against the development of post-acute Covid-19 syndrome in children and adolescents (5 to 17 years old).	Cohort study	480,298 children aged 5 to 11 and 557,638 pre-adolescents and adolescents aged 12 to 17 were analyzed. Of this sample, 88% of participants were immunized with at least two doses and 67% with at least one dose of the vaccine. For protection against probable LC symptoms, the protective capacity of the vaccine was 48.2% effective among children and 56.1% among adolescents. The sex and comorbidities of participants with LC were not analyzed separately. Among fully immunized participants, for up to 12 months, the protection rate reached 45% against probable LC cases. However, the protective factor decreased with the advancement of time when compared to the protection rates against LC between six and twelve months after immunization.	Fatigue, gastrointestinal symptoms, psychological symptoms (mental confusion), persistent systemic pain.

The review showed that the most frequently recorded LC symptoms in children and adolescents were fatigue (10 studies), headache (9), cough (9) and behavioral changes such as psychological symptoms, sleep disorders (8), mood swings, anxiety/depression (7), and difficulty concentrating (7). Other symptoms still frequently present were dyspnea (6), asthenia (3), fever (3), weight loss (5), loss of appetite (2), dysgeusia (5), dysosmia (5), abdominal pain and gastrointestinal symptoms (8), such as diarrhea (4), nausea (4), vomiting (3) and constipation (1)^(9,15,17,18,19,20,21,23,24,25,27,28,29)^.

It is worth noting that children aged 0 to 4 years tend to have a higher frequency of nasal secretion production and cough, symptoms that affect respiratory organs^(22,28)^. Children aged 5 years or older and especially adolescents aged 11 to 18 years had more occurrences of emotional instability, reduced concentration, sleep disorders, anxiety and depression, fatigue, headache, dyspnea, and muscle pain^(9,15,17, 20, 25,27)^. Furthermore, four studies showed an increase in the frequency of LC symptoms as age increases^(9,19,20,24)^.

The analysis performed comparing the sexes of the patients was divergent among the studies, making it indeterminate, with three studies showing a predominance of LC diagnosis among female children^(15,17,21)^, while an analysis signaled a predominance in the male sex^(9)^. Furthermore, one study found no significant difference between the clinical manifestations of LC presented in both sexes^(28)^. However, none of the studies analyzed the relationship between childhood LC and sex.

In this context, only one of the studies evaluated the protection of the Covid-19 vaccine against LC and it showed that there is temporary protection against the likelihood of developing the condition^(30)^. Four of the studies also analyzed a higher frequency of comorbidities among children and adolescents with LC, such as obesity, respiratory, neurological (such as epilepsy), and renal diseases^(15,19,20,27)^. However, the association of comorbidities and the development of LC was not evaluated by any of the studies.

## DISCUSSION

A symptomatic diversity of LC in children and adolescents was evidenced. In this context, physical and psychosocial symptoms were present and manifested differently among children and adolescents. Furthermore, studies differed in the incidence of LC by sex, leading to inconclusive results, and secondary diagnoses, such as obesity, were shown to be important in the susceptibility of developing LC. It is worth noting that only one study addressed the analysis of vaccination against Covid-19, pointing it as a protective factor.

LC is a multifaceted condition that affects numerous systems of the human body, employing varied pathophysiological mechanisms. Long-lasting exhaustion is one of the most common symptoms in LC, and its perpetuation may possibly be associated with toxicity in brain tissue due to the accumulation of cerebrospinal fluid caused by lymphatic drainage in these organs, in addition to changes in mitochondrial function in muscles and a decline in brain metabolism. Furthermore, tissue inflammation and structural injuries, especially if caused by insufficient oxygen and vascularization/microvascularization during the acute phase of Covid-19, are linked to damage to the cardiac system^(31)^.

Persistent inflammation and the aforementioned micro- injuries are not exclusive to the cardiac system, but are also observed in the respiratory, musculoskeletal and integumentary systems, and may promote the development of autoimmune diseases. In the immune system, the panorama is similar, fueled by chronic inflammation and having its balance disturbed, the cells of this system can undergo functional changes, such as the activation of mast cells and prolonged activation of T cells^(31)^.

Injuries acquired in the acute phase associated with this chronic inflammation process can result in long-term functional impairment in organs and oxidative stress, exacerbated by damage to the cardiac-circulatory system. In addition, other damaging mechanisms are being investigated in the kidneys and gastrointestinal system^(31)^.

In view of this context, regarding the manifestation of LC symptoms analyzed by sex, the results obtained in this review were inconclusive, due to the divergence brought by the studies. Concomitantly, in the general population, there is a greater risk of LC development in females^(3)^. The higher prevalence of LC diagnosis in women can be attributed to the fact that they frequently seek health services more for routine health care. Moreover, another explanation may lie in sex-based immunological differences, which also contribute to variations in the incidence of autoimmune diseases, which are more common in women. These factors probably explain the higher prevalence of LC in adult women, reinforcing the hypothesis that sex hormones and their immunomodulatory activity may play an important role in the development of LC in adults^(32)^.

Similarly, there were differences in the manifestation of LC symptoms by age group during LC. Children aged 0 to 4 years with LC, for example, had more respiratory complaints, while those aged 5 years or older had more psychological complaints. The physical clinical picture of both the acute disease and LC in younger children tends to be less expressive and can be explained by physiological mechanisms such as the functioning thymus organ in children up to approximately 12 years of age, in addition to the expressive deficiency of receptors of the biological catalyst that converts Angiotensin II (ACE2), vaccination status, and a significantly responsive innate immune system^(17,28,33,34)^.

Therefore, in relation to physical symptoms, the higher levels of ACE2 and its strategic location, the involvement and exposure to seasonal coronaviruses and other viral agents, combined with the cross-reactivity of immunizing agents, can cause a restriction of the inflammatory response through the performance of the opposite signaling. This way, LC symptoms can be avoided, even though the body becomes more susceptible to the infection itself^(33,34)^.

In contrast, the age threshold for LC diagnosis is defined differently in the various selected studies. For example, one study shows a higher incidence of LC among adolescents with an average age of 13 years and another study at 6 years^(15,20)^. Thus, it can be inferred that the distinctions in the prevalence of LC in children and adolescents vary according to the study, probably due to factors that may be related to the severity of the initial infection as well as their history of preexisting conditions^(1)^.

The symptomatic manifestations of LC were extensive, varied, and expressive. Therefore, physical isolation, as well as other sanitary measures required to contain the global health emergency caused by the pandemic, may have impacted children and adolescents’ clinical conditions, favoring the appearance of symptoms such as anxiety, depression, mood swings, and sleep disorders^(15,22,24,28)^. It is worth noting that many physical, psychological, and behavioral symptoms are not exclusive to LC, making it difficult for healthcare professionals to associate them with conditions that occurred after viral infection^(1)^.

With the aim of mapping symptoms and epidemiological profile, it was found that the presence of comorbidities and health conditions, biological factors and symptoms of Covid-19 infection in the acute phase can interfere in the clinical outcome of the development of LC and increase the risk of diagnosis by up to three times. Some examples are obesity, epilepsy, respiratory, neurophysiological, renal diseases and muscle pain during acute infection^(1,35-37)^.

Obesity was one of the most cited diseases in the selected studies. It is observed that the impairment of the immune system and the diagnosis of obesity favor the establishment of LC symptoms due to the damage of the acute infection itself. During the acute phase of Covid-19, there is a greater presence of pathophysiological mechanisms in patients with secondary diagnoses, mainly exacerbated inflammatory reaction. Obesity and SARS-CoV-2 infection stimulate a significant inflammatory environment, and in obesity, there is the emission of inflammatory factors, mainly Tumor Necrosis Factor Alpha (TNFa) (related to insulin resistance and diabetes) and interleukins 1 and 6 by adipose tissue. In SARS-CoV-2 infection, the inflammatory state is associated with high levels of cytokines, especially interleukins 2 and 7, MCP1, TNFa, Granulocyte Colony Stimulating Factor (G-CSF), and chemokine CXCL10^(38,39)^.

In this context, SARS-CoV-2 stimulates a plurality of inflammatory and pro-inflammatory events that reach the respiratory, neurological (microglial cells, astrocytes, brain stem), and cardiovascular systems. Thus, the organism becomes hyper- responsive to the secretion of cytokines and chemokines stimulated by the virus, favoring long-lasting symptoms. Nowadays, fatigue, the most common symptom in LC diagnoses, can also be influenced by chronic brain inflammation. Other associated processes, such as neuromuscular deterioration and psychological conditions resulting from the pandemic, are hypotheses considered^(40,41)^.

In the analysis of the relationship between vaccination status and LC, it is highlighted that the vaccine against Covid-19, in addition to acting to reduce the chances of developing the acute disease in its severe form, also promotes late protection by reducing the presence of persistent symptoms. Therefore, not only can the probabilities of infection be suppressed, but also its complexity^(30)^.

This information corroborates a Brazilian study that showed a higher incidence of LC in unvaccinated people (72%) than vaccinated people (59%), highlighting the importance of immunization strategies and maintenance of the vaccination schedule^(42)^. In Brazil, vaccines against Covid-19 are part of the pediatric vaccination schedule, consisting of two applications, at six and seven months of age, with a four-week interval between them. If the child has some compromised immune system, the schedule is carried out in three doses and if it has not been started by the pre-established age range, it can be introduced up until the child is five years old^(43)^.

Furthermore, the diagnostic process of LC involves complex clinical reasoning due to the absence of a specific test that accurately identifies biological markers related to the condition^(40)^. The importance of reports from caregivers and family members for the identification of LC, especially in children, is highlighted, in addition to the confirmation of previous Covid-19 infection through diagnostic testing associated with physical examination, blood and lung function tests, among others^(9,15,17,20,23,27,29,44)^.

Thus, extensive symptoms can compromise the physical and psychosocial well-being of children and adolescents, in addition to deteriorating their quality of life and daily activities at a fundamental stage of development. Finally, it is essential to carry out continuous monitoring of symptoms, in addition to carrying out studies that directly contribute to clinical practice, to favor the identification of cases and treatments for LC in children and adolescents. It is also important to delve deeper into the scope of clinical conditions and previous diseases that may predispose to the diagnosis of LC, seeking to understand the common biological processes and how they can worsen the clinical condition of children and adolescents.

Limitations of this review include the selection of studies in Portuguese, English and Spanish, the inclusion of articles available in full and the exclusion of indexing databases not covered in the research. However, this study contributes to the identification of the main symptoms of LC manifested in children and adolescents. With specific data on LC in the child and youth population, it becomes possible to develop more effective public health strategies, focused on prevention through encouragement of vaccination against Covid-19, early diagnosis, and appropriate treatments. Thus, understanding LC cases in children is essential not only to provide appropriate LC treatment but also to improve their quality of life.

## CONCLUSION

This study showed variability in the symptoms of LC in children and adolescents, with a higher frequency of nasal secretion and cough observed in children aged 0 to 4 years, while in adolescents aged 11 to 18 years a higher occurrence of emotional instability, reduced concentration, sleep disorders, anxiety and depression, fatigue, headache, dyspnea and muscle pain was observed. The analysis by sex was inconclusive due to the divergence of results found in the selected articles. Vaccination against Covid-19 appears to be a protective factor for LC.

Consequently, vaccination is the most effective preventive measure against childhood LC, reinforcing the need to encourage it in clinical practice and prioritize it in global and Brazilian public health policies.

Additionally, despite the growing attention to LC in the child and adolescent population, this study found gaps that require more in-depth longitudinal investigations into LC symptoms, especially studies stratified by sex and age group. Also, it is important to carry out investigations into the correlation of comorbidities, socioeconomic and environmental conditions and the consequences for the development of LC.

## DATA AVAILABILITY

The entire data set supporting the results of this study was published in the article itself.
