# Supplementary material for: Symptoms of long COVID in children and adolescents: a scoping review
Source: Rev Esc Enferm USP. 2025 Aug 4;59:e20240435. doi: 10.1590/1980-220X-REEUSP-2024-0435en (PMC12330226; doi:10.1590/1980-220X-REEUSP-2024-0435en)
Supplement: Translation

# Translation: Sintomas de COVID longa em crianças e adolescentes: uma revisão de escopo

**DOI:** 10.1590/1980-220X-REEUSP-2024-0435pt

**Authors:** Ariel Courbassier Santos de Gusmão, Ana Cristina Ribeiro La Scaléa, Silvia Carla da Silva André Uehara

**Affiliations:** 1 Universidade Federal de São Carlos, Departamento de Enfermagem, São Carlos, SP, Brasil.

**Keywords:** Criança, Adolescente, Síndrome de Pós-COVID-19 Aguda, COVID-19, Sinais e Sintomas

## Abstract

**Objetivo:**

Mapear os sintomas de Covid Longa (CL) apresentados por crianças e adolescentes.

**Método:**

Trata-se de uma revisão de escopo, utilizando os locais de busca Web of Science, Scopus, Biblioteca Virtual em Saúde e PUBMED, seguindo os princípios *Joanna Briggs Institute*.

**Resultados:**

Foram selecionados 16 estudos que mostraram que fadiga, cefaleia, dispneia e tosse foram os sintomas mais frequentes de CL. Há uma tendência do desenvolvimento de CL infanto-juvenil relacionada ao aumento da faixa etária e a correlação entre CL e sexo predominante mostrou-se inconclusiva. A presença de comorbidades como obesidade, doenças respiratórias, neurológicas e renais foi a mais relatada e um estudo mostrou uma associação entre a proteção da vacina da Covid-19 e CL.

**Conclusão:**

Esta revisão aponta para uma pluralidade de manifestações sintomatológicas da CL em crianças e adolescentes, modificando-se conforme a faixa etária e histórico de saúde.

## INTRODUÇÃO

Após 5 anos do início da pandemia, verifica-se uma endemização da doença Covid-19 e uma elevada frequência de sintomas de Covid Longa (CL) na população, incluindo crianças e adolescentes^(1)^. A CL ou condição pós-Covid são termos adotados para descrever a sintomatologia persistente, adquirida após a infecção prévia pela Covid-19 e caracterizada pela presença de sintomas após três meses a partir do seu princípio e pela continuidade por pelo menos dois meses e por não poder ser esclarecida por outros exames e enfermidades^(2)^.

Sob essa ótica, cerca de 10 a 20% das pessoas na população geral que se contaminaram com a Covid-19 podem apresentar sintomas prolongados. Apesar de ainda não estarem esclarecidos os grupos específicos mais dispostos a evoluírem para a CL, existem evidências que mulheres e pacientes que tiveram a forma grave da Covid-19 estejam mais susceptíveis^(3)^. Assim, dos mais de 651 milhões de casos de Covid-19 notificados, bem como muitos não documentados, estima-se que no mínimo 65 milhões de pessoas desenvolveram a CL, sendo de 50 a 70% de casos com história pregressa de hospitalização, 10 a 30% sem hospitalização e 10 a 12% com vacinação^(4,5,6,7)^.

Biologicamente, a CL pode estar relacionada à resposta imunológica ou inflamatória agregada e as causas podem estar associadas à existência de desequilíbrio na flora do intestino^(8)^, danos por oxidação e proteções antioxidantes diminuídas^(9)^, indícios de mal funcionamento da atividade metabólica^(10)^ e a mecanismos de defesa imunológica, lesão das células endoteliais e da latência do SARS-CoV-2; entretanto, a patogenia não está completamente esclarecida^(11)^.

Nesse contexto, observou-se que a sintomatologia de CL entre crianças e adolescentes pode ser distinta e o sistema respiratório é o mais acometido, podendo apresentar frequentemente tosse, falta de ar e dor no peito. No entanto, também podem ser observados relatos de sintomas relacionados a distúrbios gastrointestinais, diarreia, náuseas, dificuldades de concentração, quadros de irritabilidade^(1,12)^.

Deste modo, embora a CL em crianças e adolescentes possa comprometer sua qualidade de vida, interferindo negativamente nas atividades diárias, sociais, escolares e até mesmo afetando tanto o desenvolvimento físico quanto cognitivo, há uma escassez na literatura sobre essa temática nessa população. Os sintomas da CL podem ser diferentes dos observados em adultos ou idosos e podem passar despercebidos, impactando a qualidade de vida das crianças e dificultando um diagnóstico e tratamento adequado^(1,12)^. Desse modo, com vistas a buscar as lacunas persistentes na literatura quanto à sintomatologia na população infantojuvenil, este estudo propõe-se a mapear os sintomas de CL apresentados por crianças e adolescentes.

## MÉTODO

### Tipo do Estudo

Trata-se de uma revisão de escopo (scoping review) com base nos princípios preconizados pelo *Joanna Briggs Institute* (JBI). A pesquisa seguiu as seguintes etapas: (1) identificação da questão de pesquisa, (2) identificação de estudos relevantes, (3) seleção dos estudos, (4) extração de dados, (5) agrupamento, resumo e relato dos resultados, (6) consulta, (7) análise das evidências, (8) apresentação dos dados e resultados e (9) resumo dos resultados e evidências, elaboração das conclusões^(13)^.

### Definição da Questão de Pesquisa

Para conduzir a busca na revisão, a definição da questão de pesquisa foi realizada por meio do acrônimo “PCC” (população, conceito e contexto), sendo “P” crianças e adolescentes, “C” sintomas de covid longa e “C” pós pandemia: Qual a sintomatologia de CL apresentada por crianças e adolescentes, considerando idade, sexo, comorbidades e situação vacinal no pós pandemia?

### Estratégia de Busca e Critérios de Inclusão e Exclusão

A pesquisa bibliográfica foi conduzida nas bases de dados eletrônicas: *Web of Science*, *US National Library of Medicine National Institutes of Health* PUBMED – NCB, Scopus e Biblioteca Virtual em Saúde (BVS), utilizando descritores e sinônimos referenciados no Descritor em Ciências da Saúde (DeCS) e no *Medical Subject Headings* (MeSH): criança; adolescente; covid longa; Sintomas; Síndrome Pós-covid; e, em inglês: Child, Adolescent, Long COVID, Post-Acute COVID-19 Syndrome, Symptoms (Quadro 1).

**Quadro 1:** Estratégias de busca utilizadas nas bases de dados – São Carlos, SP, Brasil, 2024.

Base de Dados	Estratégia
BIBLIOTECA VIRTUAL EM SAÚDE	(child) AND (“Long Covid”) AND (symptoms)
WEB OF SCIENCE	((((AB = (Child)) OR AB = (“Adolescent”)) AND TI = (Long COVID)) OR TI = (“Post-Acute COVID-19 Syndrome”)) AND AB = (“Symptoms”)
SCOPUS	(ABS (“Child”) OR ABS (“Adolescent”) AND ABS (“Long COVID”) OR ABS (“Post-Acute COVID-19 Syndrome”) AND ABS (“Symptoms”))
PUBMED	(((“Child”[Abstract] OR “Adolescent”[Abstract]) AND “Long Covid”[Abstract]) OR “Post-Acute COVID-19 Syndrome”[Abstract]) AND “Symptoms”[Abstract]

### Extração e Seleção dos Estudos

Os critérios de inclusão foram definidos como artigos do tipo original, publicados nos idiomas inglês, português ou espanhol, independente do ano de publicação, indexados em uma das bases citadas previamente. Os critérios de exclusão foram definidos como artigos duplicados, estudos que não estavam disponíveis na íntegra, estudos cujos títulos e resumos não se delimitavam dentro do objetivo da investigação. Além disso, os catálogos de referências dos estudos também foram consultados.

Após esta etapa, as referências foram exportadas para o aplicativo web StArt (*State of the Art through Systematic Review*), para a seleção dos estudos em dois níveis. A primeira seleção foi realizada por meio da leitura de títulos e resumos, seguida pela leitura do artigo na íntegra. Os estudos elegíveis foram recuperados na íntegra e avaliados por dois pesquisadores, individualmente. Em ambas as etapas, as divergências foram debatidas até alcançarem uma concordância.

### Elaboração da Revisão

Para a seleção dos artigos foram utilizadas as diretrizes do PRISMA-Sr (*extension for scoping reviews),* para a coleta das seguintes informações: autor, ano e local do estudo, objetivo, desenho do estudo, objetivo e principais resultados. Respondendo à pergunta norteadora desse estudo, os resultados encontrados foram organizados em quadros e textos descritivos^(14)^.

## RESULTADOS

Foram encontrados nas bases de dados 700 artigos, sendo excluídos 308 por serem duplicatas. Ao analisar os 392 selecionados, 316 foram excluídos por serem revisões e artigos de opinião. Assim, foram analisados 76 estudos, sendo que 60 foram excluídos por não responderem a questão problema, sendo selecionados 16 artigos, publicados no período de 2021 a março de 2024 (Figura 1).

**Figura 1:** Fluxograma e aplicação da seleção dos estudos segundo as diretrizes do PRISMA – PCR. São Carlos, SP, Brasil, 2025.

Destaca-se que todos os estudos foram publicados em inglês e, quanto ao tipo de estudo, 7 (43,8%) eram estudos de coorte, 4 (25%) de caso-controle, 3 (18,8%) transversais, 1 (6,2%) estudo de caso e 1 (6,2%) série de casos (Quadro 2).

**Quadro 2:** Descrição dos artigos selecionados, segundo autor, ano, local, título, objetivo, tipo de estudo e principais resultados – São Carlos, SP, Brasil, 2025.

Autor, ano e Local	Objetivo	Tipo de Estudo	Principais Resultados	Principais Sintomas
Asadi-Pooya et al., 2021, Irã^(15)^.	Reconhecer em pacientes infantis diagnosticados com Covid Longa: a taxa de ocorrência, o quadro clínico e variáveis de risco.	Estudo transversal	26 crianças da amostra manifestaram sintomas de CL, sendo 15 do sexo feminino e 11 do sexo masculino. Comorbidades e dados vacinais desse grupo não foram citados. A taxa de ocorrência da CL foi de mais de 40% em pacientes hospitalizados durante a fase aguda da Covid-19.	Astenia, exaustão, incapacidade de se exercitar, presença de secreção nas vias aéreas em excesso, tosse, artralgia, cefaleia, distúrbios do sono e mialgia.
Ashkenazi-Hoffnung et al., 2021, Israel^(16)^.	Analisar o quadro clínico de 90 indivíduos da população juvenil que apresentaram sintomas da Covid Longa.	Estudo de coorte	A proporção da amostra em relação ao sexo de 90 crianças foi de 1,4 masculino/1 feminino. Ademais, as comorbidades mais frequentes foram transtorno de déficit de atenção e hiperatividade, imunodeficiência, doença autoimune ou inflamatória, asma e transtorno de ansiedade ou depressão. Dados vacinais não foram avaliados. Ainda, foi verificada uma redução de 58,9% do rendimento diário de rotinas habituais.	Exaustão, dificuldade respiratória e dores musculares, principalmente na faixa etária de 11 a 18 anos. Ademais, zumbido no ouvido, comportamentos muito repetitivos, aumento da frequência cardíaca, tosse, tremor, dor articular, emagrecimento acima de 5% do peso inicial, manifestações clínicas do sistema digestivo, distúrbios do paladar e olfato, alopecia, cefaleia, parestesia e angina; além de déficit na concentração, déficit na memória e desordens do sono.
Roge et al., 2021, Letônia^(17)^.	Reconhecer e averiguar, no grupo infantil, os múltiplos efeitos de uma infecção longa pelo vírus SARS-COV-2 além de expor os contrastes entre a sintomatologia contínua entre a Covid e outras patologias infecciosas.	Estudo de caso controle	Na amostra classificada como a que possuía sintomas de CL, 105 eram do sexo feminino e 131 do sexo masculino. A sintomatologia variou com a idade das crianças, sendo as manifestações de vias aéreas mais comuns em crianças pequenas e bebês. Em relação às comorbidades destacam-se asma brônquica, distúrbios gastrointestinais, transtornos psiquiátricos, epilepsia, fibrose cística, alergias, dermatite atópica, anomalias congênitas, distúrbios neurológicos e urológicos.	Exaustão, cefaleia, coriza, tosse, pirexia, hiposmia/hipogeusia, sudorese noturna, inapetência, emagrecimento, vertigem, artralgia, dispneia em repouso, dispneia ao esforço físico, dores musculares, sintomas ritmo-cardíacas, odinofagia, diarreia, êmese e náuseas, aumento da frequência respiratória, alopecia, linfadenopatia, presença de sibilos pulmonares, afasia e disfagia. Ainda, foram referidos déficit na concentração, déficit de memória, déficit de atenção, oscilações de humor, estresse e sintomas de ansiedade/depressão.
Bossley et al., 2022, Reino Unido^(18)^.	Analisar o quadro clínico infantil quanto ao desenvolvimento de sintomas similares aos de Covid Longa.	Estudo de coorte	Participaram do estudo 88 crianças, sendo 61% do sexo masculino e, do grupo com sintomas prolongados, os dois eram do sexo masculino, sendo que um participantes não possuía doenças de base e o outro possuía refluxo gastroesofágico, síndrome de Ehlers-Danlos, alteração genética do cromossomo 6 e insuficiência de imunoglobulina-A. Dados vacinais não foram coletados. Para 5,5% dos participantes, os sintomas de CL foram classificados como severos e 7% graves, 12,5% eram medianos. Ainda, a gravidade da sintomatologia da Covid aguda e o desenvolvimento de CL não estavam correlacionados.	Dentre esses casos, o sintoma mais presente foi a tosse não produtiva.
Kuczborska et al., 2022, Polônia^(19)^.	Contrastar as disparidades de manifestações sintomatológicas de Covid longa em infantes imunologicamente deficientes e infantes não imunologicamente deficientes, além de avaliar a taxa de ocorrência.	Estudo de caso controle	A idade dos participantes variou de 4 meses a 17 anos, sendo no grupo com deficiência imunológica 37 do sexo masculino e 33 do feminino e no grupo com normalidade imunológica 41 do sexo masculino e 36 do sexo feminino. As comorbidades mais frequentes foram imunodeficiência, transplantados renais ou hepáticos, colite com úlceras, doenças desmielinizantes e tumores. Já no grupo sem deficiência imunológica, as enfermidades alérgicas foram as mais citadas. Em relação aos dados vacinais, 3 crianças imunodeficientes e 20 crianças imunocompetentes foram vacinadas contra a Covid-19, mas nenhuma outra análise foi realizada.	O grupo com normalidade imunológica apresentou exaustão, redução da capacidade de esforço físico, déficit de concentração e perturbações do sono. Já crianças e adolescentes em vulnerabilidade imunológica manifestaram uma maior frequência de sintomas de CL gastroenterológicos.
Baptista de Lima et al., 2023^(20)^.	Detalhar as manifestações clínicas persistentes na Covid longa e seus potenciais de risco.	Estudo de coorte	Participaram do estudo crianças e adolescentes de 0 a 18 anos, sendo 54,4% do sexo masculino. Verificou-se um aumento da probabilidade de se desenvolver CL em até 3 vezes em 12 semanas quando se há alguma comorbidade envolvida; porém, a única citada é a obesidade. A fadiga pode estar relacionada com a presença de doenças como obesidade e doenças graves subjacentes. Dados vacinais não foram coletados.	Pirexia, tosse, coriza, exaustão, disfunções do sono e fadiga.
Basra et al., 2023, Indonésia^(21)^.	Compreender o panorama sintomatológico infantil de Covid Longa.	Estudo transversal	Foram analisadas 214 crianças e adolescentes de até 18 anos. Dos 121 participantes que apresentaram sintomas persistentes de Covid, 70 eram do sexo feminino e 51 eram do sexo masculino. Dados vacinais e histórico de saúde não foram analisados.	Exaustão após a prática de exercício físico ou intelectual, aumento/diminuição do ritmo cardíaco, hipotensão, desequilíbrio do mecanismo termorregulador corpóreo, inapetência, diarreia, gastralgia, perda da habilidade de concentração em informações novas e distúrbios respiratórios
Bilu et al., 2023, Israel^(22)^.	Analisar em longo prazo (até 24 meses), a saúde psicológica de jovens que obtiveram exame PCR positivo e negativo para o vírus SARS-CoV-2 e identificar se a sintomatologia psicológica está associada ou não à infecção.	Estudo de coorte	Foram analisados 22.354 adolescentes de 12 a 17 anos de março de 2020 a março de 2021. Participantes PCR negativos obtiveram maior taxa de ocorrência de prescrição de psicofármacos e diagnósticos mentais (irritabilidade, ansiedade e depressão). Se comparados por sexo, os resultados foram similares entre si, com um destaque para os participantes masculinos com PCR negativo que apresentaram maior incidência de reconhecimento de distúrbios alimentares. Neste estudo, verificou-se que a sintomatologia mental estava associada aos efeitos da emergência sanitária da Covid-19 e não necessariamente à infecção pelo vírus SARS-CoV-2, mesmo em longo prazo. Não foram analisadas situação vacinal e comorbidades.	Irritabilidade, ansiedade e depressão.
Horikoshi et al., 2023, Japão^(23)^.	Analisar as manifestações clínicas e exames de pacientes de idade pediátrica com Covid longa.	Série de casos	Participaram 24 crianças e adolescentes de 15 anos ou menos. Autismo, TDAH, transtorno do desenvolvimento, asma, disautonomia ortostática e déficit auditivo foram doenças de base encontradas na amostra. Adicionalmente, 9 participantes receberam 2 doses da vacina contra a Covid-19.	Torpor, cefaleia, disfunções do paladar e do olfato, perda completa do paladar, alopecia, dor extra membranosa, astenia, ânsia de vômito e/ou vômito e disfunções do sono, dor extra membranosa, confusão mental, dor intramembranosa, tosse, dificuldades respiratórias, diarreia, formigamento e sintomas psicóticos.
Mancino et al., 2023, Itália^(24)^.	Analisar a sintomatologia infantil da Covid Longa, incidências e o aparecimento de indicadores de risco.	Estudo de coorte	Das 697 crianças e adolescentes selecionados para o acompanhamento, 12,7% tinham entre 0 e 4 anos, 19,3% entre 5 e 10 anos; 38,7% entre 11 e 15 anos e 45% entre 16 e 18 anos, ainda 48% eram do sexo masculino e as comorbidades citadas foram bronquiolite e sibilos anteriores, sobrepeso e obesidade. Destaca-se que 81 (11,6%) crianças apresentaram sintomas persistentes após 90 dias. Dados vacinais não foram analisados.	Fraqueza, enjoos, manifestações gastroentéricas, dificuldades respiratórias, dificuldade respiratória aos esforços, tosse, disfunções cognitivas, olfativas e do paladar, cefaleia, emagrecimento, gastralgia e déficit de concentração
Sakurada et al., 2023, Japão^(25)^.	Usar da comparação de sintomas de CL em adultos e crianças/adolescentes para elucidar os índices de absenteísmo escolar e os impactos provocados pelas alterações virais.	Estudo de caso controle	Foram analisados 452 casos de CL, sendo 398 adultos (19 anos ou mais) e 54 adolescentes de 11 a 18 anos. Dos adolescentes com CL, 31 eram do sexo feminino e 23 do sexo masculino. Somente os pacientes estudantes tiveram seus dados vacinais analisados: dos 22 estudantes, 11 receberam 1 dose de vacina contra a Covid-19, 10 receberam duas doses e apenas 1 recebeu três doses. Destaca-se que 56% (28) dos adolescentes deixaram de comparecer à escola devido aos sintomas de CL. Ademais, comorbidades não foram analisadas.	Cansaço acentuado, dores de cabeça, distúrbios do sono, do olfato e do paladar.
Saniasiaya, 2023, Malásia^(26)^.	Analisar os perfis clínicos pediátricos de enxaqueca vertiginosa subsequente à doença causada pela Covid-19.	Estudo de caso	O primeiro caso foi referente a uma criança que apresentava de três a quatro vezes por semana ataques de vertigem e náuseas, 3 meses pós infecção pelo SARS-CoV-2 e mais nenhum histórico médico prévio associado. O segundo caso foi de um adolescente com queixas diárias de cefalalgia e instabilidade postural, 6 meses pós infecção pelo SARS-CoV-2 e mais nenhum histórico médico associado. As intervenções médicas propostas para o caso número 1 foram assumir um estilo de vida equilibrado e prática atlética associados à prescrição de reforços vitamínicos. Em sua reavaliação, seu quadro clínico apresentou melhoras. As intervenções médicas propostas para o caso número 2 incluíram estilo de vida equilibrado, mas foi implementada uma prescrição medicamentosa de um comprimido diário de flunarizina 5 mg.	Enxaqueca vestibular.
Seery et al., 2023, Argentina^(27)^.	Analisar as manifestações clínicas de grande duração e variáveis de risco (margem de cerca de noventa dias) trazidas pela enfermidade causada pelo SARS-CoV-2.	Estudo de caso controle	Foram analisados 639 casos de Covid-19, sendo que 219 cuidadores relataram sintomas de CL em crianças e uma média de idade de 7 anos. A prolongação dos sintomas por mais de três meses foi relacionada à presença de doenças e disfunções subjacentes como diabetes, enfermidades nos rins e no trato respiratório, além da infecção por SARS-CoV-2 ser sintomática e estar em uma faixa etária mais elevada. Os dados vacinais não foram analisados.	Emagrecimento, mialgia, exaustão, taquicardia, dificuldade respiratória, tosse, constipação ou diarreia, êmese ou náusea, convulsões e inapetência, déficits de concentração e de memória, disgeusia, anosmia, vertigem, cefaleia, sintomas depressivos e ansiosos e oscilações de humor.
Calcaterra et al., 2024, Itália^(28)^.	Acompanhar a história clínica de pacientes em faixa etária infantil submetidos à atenção hospitalar por infecção aguda de SARS-CoV-2 para a verificação do desenvolvimento de Covid Longa.	Estudo de coorte	Participaram do estudo 167 pacientes: 55% estavam abaixo dos dois anos de idade, 30% entre 2 e 10 anos e 14,3% acima de 10 anos, ainda 46,1% eram do sexo feminino e 53,9% do sexo masculino. Entretanto, os autores fazem uma conclusão afirmando que não há necessariamente uma maior prevalência em determinado sexo. As comorbidades mais presentes foram condições neurológicas, doenças gastrointestinais, condições genéticas, diabetes, doença renal, malformações cardíacas, obesidade, desnutrição crônica, asma, doenças autoimunes, tumores e outros. Dados vacinais não foram analisados.	Emagrecimento, insônia, sibilos pulmonares, gastralgia, fadiga, tosse persistente, falta de apetite, mialgias, cefaleias, artralgia e oscilações de humor.
Meiere et al., 2024, Letônia^(29)^.	Analisar faixas etárias infantis do diagnóstico de Covid Longa em pelo menos 30 dias seguidamente à infecção primária por SARS-CoV-2.	Estudo transversal	A amostra foi de 220 participantes menores de 18 anos, 56,8% pertenciam ao sexo feminino e 43,2% ao sexo masculino. Dados vacinais e comorbidades não foram analisados.	Epigastralgia, cansaço extremo, cefaleia, oscilações de humor, restrição da capacidade física, sonolência, tosse produtiva, rinorreia, déficit de memória, taquicardia e angina.
Razzaghi et al., 2024, Estados Unidos^(30)^.	Mensurar em infantojuvenis (5 a 17 anos) o resultado da proteção vacinal contra o desenvolvimento da síndrome pós aguda da Covid-19.	Estudo de coorte	Foram analisadas 480.298 crianças de 5 a 11 anos e 557.638 pré-adolescentes e adolescentes de 12 a 17 anos. Dessa amostra, 88% dos participantes foram imunizados com no mínimo duas doses e 67% com pelo menos uma dose da vacina. Para proteção contra provável sintomatologia de CL, a capacidade protetora da vacina teve eficácia de 48,2% entre crianças e 56,1% entre adolescentes. Não foram analisados isoladamente o sexo e comorbidades dos participantes com CL. Entre participantes totalmente imunizados, por até 12 meses, a taxa de proteção atingiu 45% contra prováveis casos de CL. Entretanto, o fator protetivo apresentou redução com o avanço do tempo quando comparadas às taxas de proteção contra CL entre seis e doze meses posterior à imunização.	Fadiga, sintomas gastrointestinais, sintomas psicológicos (confusão mental), dor sistêmica persistente.

A revisão mostrou que os sintomas de CL registrados com maior frequência em crianças e adolescentes foram fadiga (10 estudos), cefaleia (9), tosse (9) e modificações de conduta como sintomas psicológicos, transtornos do sono (8), oscilações de humor, ansiedade/depressão (7) e dificuldade de concentração (7). Outros sintomas ainda presentes com frequência foram dispneia (6), astenia (3), febre (3), emagrecimento (5), inapetência (2), disgeusia (5), disosmia (5), dor abdominal e sintomas gastrointestinais (8), como diarreia (4), náuseas (4), vômitos (3) e constipação (1)^(9,15,17,18,19,20,21,23,24,25,27,28,29)^.

Destaca-se que crianças de 0 a 4 anos tendem a apresentar uma maior frequência de produção de secreção nasal e tosse, sintomas que acometem órgãos respiratórios^(22,28)^. Já crianças de 5 anos ou mais e especialmente adolescentes de 11 a 18 anos tiveram mais ocorrências de instabilidade emocional, concentração reduzida, distúrbios do sono, ansiedade e depressão, fadiga, cefaleia, dispneia e dores musculares^(9,15,17,20,25,27)^. Além disso, quatro estudos mostraram um aumento da frequência de sintomas de CL conforme aumenta a faixa etária^(9,19,20,24)^.

A análise realizada comparando os sexos dos pacientes foi divergente entre os estudos, tornando-a indeterminada, sendo que três estudos apresentaram uma predominância do diagnóstico de CL entre crianças do sexo feminino^(15,17,21)^, enquanto uma análise sinalizou a predominância no sexo masculino^(9)^. Além disso, um estudo não encontrou diferença significativa entre as manifestações clínicas de CL apresentadas em ambos os sexos^(28)^. No entanto, nenhum dos estudos analisou a relação entre a CL infantojuvenil e o sexo.

Nesse contexto, apenas um dos estudos avaliou a proteção da vacina da Covid-19 contra a CL e ele mostrou que existe uma proteção temporária contra a probabilidade de desenvolvimento da condição^(30)^. Quatro dos estudos também analisaram uma maior frequência de comorbidades entre pacientes infanto-juvenis com CL, como obesidade, doenças respiratórias, neurológicas (como epilepsia) e renais^(15,19,20,27)^. Entretanto, a associação das comorbidades e o desenvolvimento de CL não foi avaliada por nenhum dos estudos.

## DISCUSSÃO

Foi evidenciada uma diversidade sintomatológica de CL em crianças e adolescentes. Nesse contexto, sintomas físicos e psicossociais estavam presentes e manifestaram diferentemente entre crianças e adolescentes. Além disso, estudos divergiram na incidência da CL por sexo, tornando os resultados inconclusivos e diagnósticos secundários, como obesidade, mostraram-se importantes na susceptibilidade do desenvolvimento da CL. Cabe destacar que apenas um estudo abordou a análise da vacinação contra a Covid-19, apontando-a como um fator de proteção.

A CL é uma condição multifacetada que acomete numerosos sistemas do corpo humano, empregando variados mecanismos fisiopatológicos. A exaustão duradoura é um dos sintomas mais presentes em quadros de CL, podendo ter sua perpetuação possivelmente associada à toxicidade nos tecidos cerebrais devido ao acúmulo de líquido cefalorraquidiano ocasionado pela drenagem linfática nesses órgãos, além das alterações na função mitocondrial nos músculos e declínio do metabolismo encefálico. Outrossim, a inflamação tecidual e ferimentos estruturais, especialmente se causados por insuficiência de oxigênio e vascularização/microvascularização durante a fase aguda da Covid-19, estão atrelados ao prejuízo do sistema cardíaco^(31)^.

A inflamação persistente e os microferimentos supracitados não são exclusivos do sistema cardíaco, sendo observados também no sistema respiratório, osteomuscular e tegumentar, podendo fomentar o desenvolvimento de doenças autoimunes. No sistema imunológico, o panorama é semelhante, fomentado pela inflamação crônica e tendo seu equilíbrio perturbado, as células desse sistema podem sofrer alterações funcionais, como na ativação dos mastócitos e ativação prolongada das células T^(31)^.

As lesões adquiridas na fase aguda associadas a esse processo de inflamação crônica podem resultar em prejuízo funcional de longo prazo nos órgãos e estresse oxidativo, potencializados pelos danos no sistema cardíaco-circulatório. Além disso, outros mecanismos danosos estão sendo investigados nos rins e no sistema gastrointestinal^(31)^.

Tendo em vista esse contexto, quanto à manifestação dos sintomas de CL analisada por sexo, os resultados obtidos nesta revisão foram inconclusivos, devido à divergência trazida pelos estudos. Paralelamente, na população geral, há um maior risco de desenvolvimento de CL em pessoas do sexo feminino^(3)^. A maior prevalência de diagnóstico de CL em mulheres pode ser atribuída ao fato de que elas frequentemente buscam mais os serviços de saúde por cuidados rotineiros à saúde. Além disso, outra explicação pode estar nas diferenças imunológicas baseadas no sexo, que também contribuem para as variações na incidência de doenças autoimunes, que são mais comuns em mulheres. Esses fatores provavelmente explicam a prevalência maior de CL em mulheres adultas, reforçando a hipótese de que os hormônios sexuais e sua atividade imunomoduladora podem desempenhar um papel importante no desenvolvimento da CL em adultos^(32)^.

Analogamente, houve diferenças na manifestação dos sintomas de CL por faixa etária durante a CL. As crianças de 0 a 4 anos com CL, por exemplo, manifestaram mais queixas respiratórias, enquanto as de 5 anos ou mais tiveram mais queixas psicológicas. O quadro clínico físico tanto da doença aguda como da CL de crianças mais novas tende a ser menos expressivo e pode ser explicado por mecanismos fisiológicos como o órgão do timo operante em crianças de até aproximadamente 12 anos, além da deficiência expressiva de receptores do catalisador biológico que converte a Angiotensina II (ACE2), situação vacinal e um sistema imune inato significativamente responsivo^(17,28,33,34)^.

Assim, em relação aos sintomas físicos, os maiores níveis de ACE2 e sua localização estratégica, o envolvimento e exposição a coronavírus estacionais e outros agentes virais, aliados à reatividade cruzada dos agentes imunizantes, podem ocasionar uma restrição da resposta inflamatória a partir da realização da sinalização contrária. Desta forma, os sintomas de CL podem ser evitados, ainda que o organismo se torne mais suscetível à infecção em si^(33,34)^.

Em contrapartida, o limiar da faixa etária do diagnóstico de CL é definido de forma distinta nos vários estudos selecionados. Por exemplo, um dos estudos mostra uma maior incidência de CL entre adolescentes com idade média de 13 anos e outro estudo aos 6 anos^(15,20)^. Deste modo, pode-se inferir que as distinções na prevalência de CL em crianças e adolescentes variam conforme o estudo, provavelmente devido a fatores que podem estar relacionados à gravidade da infecção inicial bem como seu histórico de condições preexistentes^(1)^.

As manifestações sintomatológicas de CL mostraram-se extensas, variadas e expressivas. Destarte, o isolamento físico, igualmente outras medidas sanitárias necessárias para conter a emergência global de saúde ocasionada pela pandemia, podem ter impactado em quadros clínicos infanto-juvenis, favorecendo o aparecimento de sintomas como ansiedade, depressão, oscilação de humor e distúrbios do sono^(15,22,24,28)^. Cabe destacar que muitos sintomas físicos, psicológicos e comportamentais não são exclusivos da CL, dificultando a sua associação pelos profissionais da saúde a condições que ocorreram pós infecção viral^(1)^.

Sob o objetivo de mapeamento de sintomas e perfil epidemiológico, foi encontrado que a presença de comorbidades e condições de saúde, fatores biológicos e sintomatologia da infecção por Covid-19 na fase aguda podem interferir no desfecho clínico do desenvolvimento da CL e aumentar o risco do diagnóstico em até três vezes. Alguns exemplos são obesidade, epilepsia, doenças respiratórias, neurofisiológicas, renais e dores musculares durante a infecção aguda^(1,35,36,37)^.

A obesidade foi uma das doenças mais citadas nos estudos selecionados. Observa-se que o comprometimento do sistema imunológico e o diagnóstico de obesidade favorecem o estabelecimento de sintomas de CL devido ao prejuízo da própria infecção aguda. Durante a fase aguda da Covid-19, verifica-se uma maior presença de mecanismos fisiopatológicos em pacientes com diagnósticos secundários, principalmente reação inflamatória exacerbada. A obesidade e a infecção por SARS-CoV-2 estimulam um ambiente inflamatório significativo, sendo que, na obesidade, ocorre a emissão de fatores inflamatórios, principalmente o Fator de Necrose Tumoral Alfa (TNFa) (relacionado com a resistência insulínica e diabetes) e interleucinas 1 e 6 pelo tecido adiposo. Já na infecção pelo SARS-CoV-2, o estado inflamatório está associado aos elevados níveis de citocinas, especialmente interleucinas 2 e 7, MCP1, TNFa, Fator Estimulador de Colônias de Granulócitos (G-CSF) e quimiocina CXCL10^(38,39)^.

Nesse contexto, o SARS-CoV-2 estimula uma pluralidade de eventos inflamatórios e pró- inflamatórios que alcançam os sistemas respiratório, neurológico (células da microglia, astrócitos, tronco cerebral) e cardiovascular. Desta forma, o organismo torna-se hiper responsivo a secreção de citocinas e quimiocinas estimuladas pelo vírus, favorecendo sintomas de longa duração. Hodiernamente, a fadiga, sintoma mais recorrente nos diagnósticos de CL, também pode ser influenciada pela inflamação crônica cerebral. Outros processos aliados, como deteriorações neuromusculares e condições psicológicas resultantes da pandemia, são hipóteses consideradas^(40,41)^.

Na análise sobre a relação entre situação vacinal e CL, destaca-se que a vacina contra a Covid-19, além de atuar na redução das chances de desenvolver a doença aguda em sua forma grave, promove também proteções tardias por meio da diminuição da presença de sintomas persistentes. Portanto, não somente as probabilidades de infecção podem ser suprimidas, mas também a sua complexidade^(30)^.

Essa informação corrobora um estudo brasileiro que mostrou uma incidência de CL maior em pessoas não vacinadas (72%) do que vacinadas (59%), colocando em evidência a importância das estratégias de imunização e manutenção do esquema vacinal^(42)^. No Brasil, as vacinas contra a Covid-19 fazem parte do esquema vacinal pediátrico, sendo composto por duas aplicações, aos seis e sete meses de idade, com quatro semanas de intervalo entre elas. Caso a criança possua algum comprometimento do sistema imune, o esquema é realizado em três doses e caso não tenha sido iniciado até a faixa etária pré-estabelecida, pode ser introduzido até os cinco anos incompletos^(43)^.

Ainda, o processo diagnóstico da CL envolve um raciocínio clínico complexo devido à ausência de um exame específico que identifique com precisão marcadores biológicos relacionados ao quadro^(40)^. Ressalta-se a importância dos relatos dos cuidadores e familiares para a identificação da CL, especialmente em crianças, além da confirmação de infecção anterior por Covid-19 por meio de teste diagnóstico associado a exame físico, sanguíneo, função pulmonar, entre outros^(9,15,17,20,23,27,29,44)^.

Assim, a extensa sintomatologia pode comprometer o bem estar físico e psicossocial de crianças e adolescentes, além de deteriorar sua qualidade de vida e atividades diárias em uma fase fundamental de desenvolvimento. Por fim, é fundamental realizar um monitoramento contínuo dos sintomas, além de ser imprescindível a realização de estudos que contribuam diretamente na prática clínica, de forma a favorecer a identificação dos casos e tratamentos para a CL em crianças e adolescentes. Também é importante o aprofundamento no âmbito das condições clínicas e doenças prévias que podem predispor ao diagnóstico de CL, buscando a compreensão dos processos biológicos em comum e de que forma pode agravar o quadro clínico infanto-juvenil.

Consideram-se como limitações desta revisão a seleção de estudos em português, inglês e espanhol, a inclusão de artigos disponíveis na íntegra e a exclusão de bases de indexação não contempladas na pesquisa. No entanto, este estudo contribui para a identificação dos principais sintomas de CL manifestados em crianças e adolescentes. Com dados específicos sobre a CL na população infanto juvenil, torna-se possível elaborar estratégias de saúde pública mais eficazes, focadas na prevenção por meio do incentivo à vacinação contra a Covid-19, no diagnóstico precoce e em tratamentos adequados. Assim, compreender os casos de CL em crianças é essencial não apenas para proporcionar o tratamento apropriado da CL, mas também para melhorar sua qualidade de vida.

## CONCLUSÃO

Este estudo evidenciou uma variabilidade na sintomatologia da CL em crianças e adolescentes, sendo observada uma maior frequência de produção de secreção nasal e tosse em crianças de 0 a 4 anos, enquanto em adolescentes de 11 a 18 anos foi observada uma maior ocorrência de instabilidade emocional, concentração reduzida, distúrbios do sono, ansiedade e depressão, fadiga, cefaleia, dispneia e dores musculares. A análise por sexo foi inconclusiva devido à divergência de resultados encontrada nos artigos selecionados. A vacinação contra a Covid-19 apresenta-se como um fator protetor para a CL.

Deste modo, a vacinação apresenta-se como a medida de prevenção mais eficaz contra a CL infantojuvenil, reforçando a necessidade de incentivá-la na prática clínica e priorizá-la nas políticas de saúde públicas mundiais e brasileiras.

Ademais, apesar da crescente atenção à CL na população infantojuvenil, esse estudo identificou lacunas que carecem de investigações longitudinais e mais aprofundadas sobre os sintomas de CL, principalmente de estudos estratificados por sexo e faixa etária. Ainda, é importante a realização de investigações sobre a correlação de comorbidades, condições socioeconômicas e ambientais e as consequências para o desenvolvimento da CL.

## DISPONIBILIDADE DE DADOS

Todo o conjunto de dados que dá suporte aos resultados deste estudo foi publicado no próprio artigo.

# Translation: Síntomas de COVID largo en niños y adolescentes: una revisión de alcance

**DOI:** 10.1590/1980-220X-REEUSP-2024-0435es

**Authors:** Ariel Courbassier Santos de Gusmão, Ana Cristina Ribeiro La Scaléa, Silvia Carla da Silva André Uehara

**Affiliations:** 1 Universidade Federal de São Carlos, Departamento de Enfermagem, São Carlos, SP, Brasil.

**Keywords:** Niño, Adolescente, Síndrome Post Agudo de COVID-19, COVID-19, Signos y Síntomas

## Abstract

**Objetivo:**

Mapear los síntomas de Covid Prolongada (CP) que presentan niños y adolescentes.

**Método:**

Se trata de una revisión del alcance, que utiliza los motores de búsqueda Web of Science, Scopus, Virtual Health Library y PUBMED, siguiendo los principios *Joanna Briggs Institute*.

**Resultados:**

Se seleccionaron 16 estudios que mostraron que la fatiga, el dolor de cabeza, la disnea y la tos eran los síntomas más frecuentes de CP. Existe una tendencia al desarrollo de CP infanto-juvenil relacionada con el aumento del rango de edad y la correlación entre CP y sexo predominante resultó no concluyente. La presencia de comorbilidades como obesidad, enfermedades respiratorias, neurológicas y renales fue la más reportada y un estudio mostró una asociación entre la protección de la vacuna contra la Covid-19 y la CP.

**Conclusión:**

Esta revisión señala una pluralidad de manifestaciones sintomáticas de CP en niños y adolescentes, que cambian según el grupo de edad y el historial de salud.

## INTRODUCCIÓN

Cinco años después del inicio de la pandemia, se ha producido una propagación endémica de la enfermedad Covid-19 y una alta frecuencia de síntomas de Covid largo (CL) en la población, incluidos niños y adolescentes^(1)^. CL o afección post- Covid son términos utilizados para describir síntomas persistentes adquiridos tras una infección previa por Covid-19 y caracterizados por la presencia de síntomas tres meses después de su inicio y que continúan durante al menos dos meses, y porque no pueden aclararse mediante otras pruebas y enfermedades^(2)^.

Desde este punto de vista, entre el 10% y el 20% de las personas de la población general que se han infectado con Covid-19 pueden experimentar síntomas prolongados. Aunque aún no están claros los grupos específicos con más probabilidades de desarrollar CL, existen pruebas de que las mujeres y los pacientes que han padecido la forma grave de Covid-19 son más susceptibles^(3)^. Por tanto, de los más de 651 millones de casos de Covid-19 notificados, además de muchos no documentados, se estima que al menos 65 millones de personas han desarrollado CL, con un 50 a 70% de casos con antecedentes de hospitalización, un 10 a 30% sin hospitalización y un 10 a 12% con vacunación^(4,5,6,7)^.

Biológicamente, CL puede estar relacionada con una respuesta inmunitaria o inflamatoria agregada y las causas pueden estar asociadas a un desequilibrio de la flora intestinal^(8)^, daño oxidativo y protección antioxidante reducida^(9)^, evidencias de un mal funcionamiento de la actividad metabólica^(10)^ y de los mecanismos de defensa inmunitaria, daños en las células endoteliales y latencia del SARS-CoV-2; sin embargo, la patogenia no se conoce del todo^(11)^.

En este contexto, se ha observado que los síntomas de CL entre niños y adolescentes pueden ser distintos y que el sistema respiratorio es el más afectado, con tos frecuente, dificultad para respirar y dolor torácico. Sin embargo, también se han notificado síntomas relacionados con trastornos gastrointestinales, diarrea, náuseas, dificultad para concentrarse e irritabilidad^(1,12)^.

Por lo tanto, a pesar de que CL en niños y adolescentes puede comprometer su calidad de vida, interfiriendo negativamente en las actividades cotidianas, sociales y escolares e incluso afectando el desarrollo tanto físico como cognitivo, existe escasa literatura sobre el tema en esta población. Los síntomas de CL pueden ser diferentes de los observados en adultos o ancianos y pueden pasar desapercibidos, repercutiendo en la calidad de vida de los niños y dificultando su diagnóstico y tratamiento adecuados^(1,12)^. Por lo tanto, con el fin de buscar las lagunas persistentes en la literatura en relación con los síntomas en niños y adolescentes, este estudio tiene como objetivo mapear los síntomas de CL presentados por niños y adolescentes.

## MÉTODO

### Tipo de Estudio

Se trata de una revisión de alcance, basada en los principios propugnados *por el Instituto Joanna Briggs* (JBI). La investigación ha seguido las siguientes etapas: (1) identificación de la pregunta de investigación, (2) identificación de los estudios pertinentes, (3) selección de los estudios, (4) extracción de los datos, (5) agrupación, resumen y comunicación de los resultados, (6) consulta, (7) análisis de las pruebas, (8) presentación de los datos y los resultados y (9) resumen de los resultados y las pruebas, elaboración de las conclusiones^(13)^.

### Definición de la Pregunta de Investigación

Para llevar a cabo la búsqueda en la revisión, la pregunta de investigación se definió utilizando el acrónimo “PCC” (población, concepto y contexto), siendo “P” niños y adolescentes, “C” síntomas de COVID larga y “C” postpandémica: ¿Cuáles son los síntomas de CL que presentan los niños y adolescentes, teniendo en cuenta la edad, el sexo, las comorbilidades y el estado de vacunación en el periodo postpandémico?

### Estrategia de Búsqueda y Criterios de Inclusión y Exclusión

La pesquisa bibliográfica fue realizada en las bases de datos electrónicas: *Web of Science*, *US National Library of Medicine National Institutes of* Health PUBMED NCB, Scopus y Virtual Health Library (BVS), utilizando descriptores y sinónimos referenciados en el Descriptor en Health Sciences (DeCS) y en los *Medical Subject Headings* (MeSH): niño; adolescente; covid largo; Síntomas; Síndrome Post-COVID; y, en inglés: Child, Adolescent, Long COVID, Post-Acute COVID-19 Syndrome, Symptoms (Cuadro 1).

**Cuadro 1:** Estrategias de búsqueda utilizadas en las bases de datos – São Carlos, SP, Brasil, 2024.

Base de Datos	Estrategia
BIBLIOTECA VIRTUAL EN SALUD	(child) AND (“Long Covid”) AND (symptoms)
WEB OF SCIENCE	((((AB = (Child)) OR AB = (“Adolescent”)) AND TI = (Long COVID)) OR TI = (“Post-Acute COVID-19 Syndrome”)) AND AB = (“Symptoms”)
SCOPUS	(ABS (“Child”) OR ABS (“Adolescent”) AND ABS (“Long COVID”) OR ABS (“Post-Acute COVID-19 Syndrome”) AND ABS (“Symptoms”))
PUBMED	(((“Child”[Abstract] OR “Adolescent”[Abstract]) AND “Long Covid”[Abstract]) OR “Post-Acute COVID-19 Syndrome”[Abstract]) AND “Symptoms”[Abstract]

### Extracción y Selección de Estudios

Los criterios de inclusión fueron definidos como artículos originales publicados en inglés, portugués o español, independientemente del año de publicación, indexados en una de las bases de datos mencionadas anteriormente. Los criterios de exclusión fueron definidos como artículos duplicados, estudios que no estuvieran disponibles en su totalidad, estudios cuyos títulos y resúmenes no estuvieran dentro del alcance de la investigación. También se consultaron los catálogos de referencia de los estudios.

Tras esta etapa, las referencias se exportaron a la aplicación web StArt (*State of the Art through Systematic Review*) para seleccionar los estudios en dos niveles. La primera selección se realizó mediante la lectura de los títulos y resúmenes, seguida de la lectura del artículo completo. Los estudios elegibles se recuperaron en su totalidad y fueron evaluados por dos investigadores, individualmente. En ambas etapas se debatieron las diferencias hasta llegar a un acuerdo.

### Elaboración de la Revisión

Para seleccionar los artículos, se utilizaron las directrices PRISMA-Sr (*extensión para revisiones de alcance*) para recoger la siguiente información: autor, año y lugar del estudio, objetivo, diseño del estudio, objetivo y principales resultados. En respuesta a la pregunta guía de este estudio, los resultados encontrados se organizaron en tablas y textos descriptivos^(14)^.

## RESULTADOS

Se encontraron 700 artículos en las bases de datos, 308 de los cuales se excluyeron por ser duplicados. Al analizar los 392 seleccionados, 316 fueron excluidos por tratarse de revisiones y artículos de opinión. Así, se analizaron 76 estudios, 60 de los cuales fueron excluidos por no responder a la pregunta problema, y se seleccionaron 16 artículos, publicados entre 2021 y marzo de 2024 (Figura 1).

**Figura 1:** Flujograma y aplicación de la selección de estudios según las directrices PRISMA-PCR. São Carlos, SP, Brasil, 2025.

Cabe señalar que todos los estudios se publicaron en inglés y, en cuanto al tipo de estudio, 7 (43,8%) eran estudios de cohortes, 4 (25%) eran estudios de casos y controles, 3 (18,8%) eran estudios transversales, 1 (6,2%) era un estudio de casos y 1 (6,2%) era una serie de casos (Cuadro 2).

**Cuadro 2:** Descripción de los artículos seleccionados, según autor, año, localización, título, objetivo, tipo de estudio y principales resultados – São Carlos, SP, Brasil, 2025.

Autor, ano e Local	Objetivo	Tipo de Estudo	Principais Resultados	Principais Sintomas
Asadi- Pooya et al., 2021, Irán^(15)^.	Reconocer en pacientes infantiles diagnosticados de Covid Larga: la tasa de aparición, el cuadro clínico y las variables de riesgo.	Estudio transversal	26 niños de la muestra presentaban síntomas de CL, de los cuales 15 eran mujeres y 11 varones. No se mencionaron las comorbilidades ni los datos de vacunación de este grupo. La tasa de aparición de CL fue superior al 40% en los pacientes hospitalizados durante la fase aguda de Covid-19.	Astenia, agotamiento, incapacidad para el ejercicio, secreción excesiva de las vías respiratorias, tos, artralgia, cefalea, trastornos del sueño y mialgia.
Ashkenazi-Hoffnung et al., 2021, Israel^(16)^.	Analizar el cuadro clínico de 90 individuos de la población juvenil que presentaron síntomas de Covid Longa.	Estudio de cohortes	La proporción de sexos de la muestra de 90 niños era de 1,4 varones/1 mujer. Además, las comorbilidades más frecuentes fueron trastorno por déficit de atención con hiperactividad, inmunodeficiencia, enfermedad autoinmune o inflamatoria, asma y ansiedad o depresión. No se evaluaron los datos de vacunación. Además, hubo una reducción del 58,9% en el rendimiento de la rutina diaria.	Agotamiento, dificultad para respirar y dolores musculares, especialmente en el grupo de edad de 11 a 18 años. Además, zumbidos en los oídos, comportamientos muy repetitivos, aumento de la frecuencia cardiaca, tos, temblores, dolores articulares, pérdida de peso superior al 5% del peso inicial, manifestaciones clínicas del aparato digestivo, trastornos del gusto y del olfato, alopecia, cefalea, parestesias y angina de pecho; así como déficit de concentración, déficit de memoria y trastornos del sueño.
Roge et al., 2021, Letonia^(17)^.	Reconocer e investigar, en el grupo infantil, los múltiples efectos de una larga infección por el virus SARS-COV-2, así como exponer los contrastes entre la sintomatología continua entre Covid y otras patologías infecciosas.	Estudio de caso de control	En la muestra clasificada con síntomas de CL, 105 eran mujeres y 131 varones. Los síntomas variaban según la edad de los niños, siendo las manifestaciones de las vías respiratorias más frecuentes en niños pequeños y bebés. Las comorbilidades incluían asma bronquial, trastornos gastrointestinales, trastornos psiquiátricos, epilepsia, fibrosis quística, alergias, dermatitis atópica, anomalías congénitas, trastornos neurológicos y urológicos.	Agotamiento, cefalea, rinorrea, tos, pirexia, hiposmia/hipogeusia, sudores nocturnos, inapetencia, pérdida de peso, vértigo, artralgia, disnea de reposo, disnea de esfuerzo, dolores musculares, síntomas del ritmo cardiaco, odinofagia, diarrea, emesis y náuseas, aumento de la frecuencia respiratoria, alopecia, linfadenopatías, sibilancias pulmonares, afasia y disfagia. También se notificaron déficits de concentración, de memoria y de atención, cambios de humor, estrés y síntomas de ansiedad/depresión.
Bossley et al., 2022, Reino Unido^(18)^.	Analizar el cuadro clínico de los niños en cuanto al desarrollo de síntomas similares a los de Covid Longa.	Estudio de cohortes	Participaron en el estudio 88 niños, el 61% de los cuales eran varones. Del grupo con síntomas prolongados, dos eran varones, uno de los cuales no padecía ninguna enfermedad subyacente y el otro padecía reflujo gastroesofágico, síndrome de Ehlers-Danlos, alteración genética del cromosoma 6 e insuficiencia de inmunoglobulina A. No se recogieron datos sobre vacunación. En el 5,5% de los participantes, los síntomas de CL se clasificaron como graves y en el 7% como graves, mientras que en el 12,5% fueron medios. Además, la gravedad de los síntomas Covid aguda y el desarrollo de CL no estaban correlacionados.	Entre estos casos, el síntoma más común era la tos no productiva.
Kuczborska et al., 2022, Polonia^(19)^.	Contrastar las disparidades en las manifestaciones sintomatológicas de Covid longa en lactantes inmunodeficientes y no inmunodeficientes, así como evaluar la tasa de aparición de.	Estudio de caso de control	La edad de los participantes oscilaba entre los 4 meses y los 17 años, con 37 varones y 33 mujeres en el grupo de inmunodeficiencia y 41 varones y 36 mujeres en el grupo de normalidad inmunitaria. Las comorbilidades más frecuentes eran la inmunodeficiencia, los trasplantes de riñón o hígado, la colitis con úlceras, las enfermedades desmielinizantes y los tumores. En el grupo sin inmunodeficiencia, las enfermedades alérgicas fueron las más mencionadas. En cuanto a los datos de vacunación, 3 niños inmunodeficientes y 20 inmunocompetentes fueron vacunados contra Covid-19, pero no se realizaron otros análisis.	El grupo inmunológicamente normal mostró agotamiento, menor capacidad de esfuerzo físico, déficit de concentración y alteraciones del sueño. Por otro lado, los niños y adolescentes con vulnerabilidad inmunológica mostraron una mayor frecuencia de síntomas gastroenterológicos de CL.
Baptista de Lima et al., 2023^(20)^.	Detallar las manifestaciones clínicas persistentes en Covid largo y su potencial de riesgo.	Estudio de cohortes	En el estudio participaron niños y adolescentes de 0 a 18 años, el 54,4% de los cuales eran varones. Se observó un aumento de la probabilidad de desarrollar CL de hasta 3 veces en 12 semanas cuando hay alguna comorbilidad implicada; sin embargo, la única citada fue la obesidad. La fatiga puede estar relacionada con la presencia de enfermedades como la obesidad y enfermedades subyacentes graves. No se recogieron datos sobre vacunación.	Pirexia, tos, secreción nasal, agotamiento, trastornos del sueño y fatiga.
Basra et al., 2023, Indonesia^(21)^.	Comprender el panorama sintomatológico de Covid Larga.	Estudio transversal	Se analizaron 214 niños y adolescentes de hasta 18 años. De los 121 participantes que presentaban síntomas persistentes de Covid, 70 eran mujeres y 51 varones. No se analizaron los datos de vacunación ni los antecedentes sanitarios.	Agotamiento tras el ejercicio físico o intelectual, aumento/disminución de la frecuencia cardiaca, hipotensión, desequilibrio del mecanismo termorregulador del organismo, inapetencia, diarrea, gastralgia, pérdida de la capacidad de concentración ante nuevas informaciones y trastornos respiratorios.
Bilu et al., 2023, Israel^(22)^.	Analizar, a largo plazo (hasta 24 meses), la salud psicológica de los jóvenes que dieron positivo y negativo en las pruebas de PCR para el virus SARS-CoV-2 e identificar si los síntomas psicológicos están o no asociados a la infección.	Estudio de la cohorte	Se analizaron 22.354 adolescentes de 12 a 17 años desde hasta marzo de 2021. Los participantes con PCR negativa tenían una mayor tasa de prescripciones de fármacos psicotrópicos y diagnósticos mentales (irritabilidad, ansiedad y depresión). Cuando se compararon por género, los resultados fueron similares, y los participantes masculinos con PCR negativa tuvieron una mayor incidencia de reconocimiento de trastornos alimentarios. Este estudio descubrió que los síntomas mentales estaban asociados a los efectos de la emergencia sanitaria Covid-19 y no necesariamente a la infección por SARS-CoV-2, incluso a largo plazo. No se analizaron el estado de vacunación ni las comorbilidades.	Irritabilidad, ansiedad y depresión.
Horikoshi et al., 2023, Japón^(23)^.	Analizar las manifestaciones clínicas y las exploraciones de los pacientes pediátricos con Covid largo.	Serie de casos	Participaron 24 niños y adolescentes menores de 15 años. Autismo, TDAH, trastornos del desarrollo, asma, disautonomía ortostática y discapacidad auditiva fueron las enfermedades subyacentes detectadas en la muestra. Además, 9 participantes recibieron 2 dosis de la vacuna Covid-19.	Somnolencia, cefalea, trastornos del gusto y del olfato, pérdida completa del gusto, alopecia, dolor extramembranoso, astenia, vómitos y/o vómitos y trastornos del sueño, confusión mental, dolor intramembranoso, tos, dificultades respiratorias, diarrea, hormigueo y síntomas psicóticos.
Mancino et al., 2023, Italia^(24)^.	Análisis de los síntomas de los niños Covid Larga, incidencias y aparición de indicadores de riesgo.	Estudio de cohortes	De los 697 niños y adolescentes seleccionados para el seguimiento, el 12,7% tenían entre 0 y 4 años, el 19,3% entre 5 y 10 años, el 38,7% entre 11 y 15 años y el 45% entre 16 y 18 años, el 48% eran varones y las comorbilidades citadas fueron bronquiolitis y sibilancias previas, sobrepeso y obesidad. Cabe destacar que 81 (11,6%) niños presentaban síntomas persistentes después de 90 días. No se analizaron los datos de vacunación.	Debilidad, náuseas, manifestaciones gastroentéricas, dificultades respiratorias, dificultad respiratoria de esfuerzo, tos, disfunciones cognitivas, olfativas y gustativas, cefalea, pérdida de peso, gastralgia y déficit de concentración.
Sakurada et al., 2023, Japón^(25)^.	Utilizar una comparación de los síntomas de CL en adultos y niños/adolescentes para dilucidar las tasas de absentismo escolar y los impactos causados por las alteraciones víricas.	Estudio de caso de control	Se analizaron un total de 452 casos de CL, de los cuales 398 eran adultos (de 19 años o más) y 54 eran adolescentes de entre 11 y 18 años. De los adolescentes con CL, 31 eran mujeres y 23 hombres. Sólo se analizaron los datos de vacunación de los pacientes estudiantes: de los 22 estudiantes, 11 recibieron una dosis de la vacuna Covid-19, 10 recibieron dos dosis y sólo 1 recibió tres dosis. Cabe destacar que el 56% (28) de los adolescentes dejaron de asistir a la escuela debido a los síntomas de CL. Además, no se analizaron las comorbilidades.	Cansancio intenso, dolores de cabeza, alteraciones del sueño, del olfato y del gusto.
Saniasiaya, 2023, Malasia^(26)^.	Analizar los perfiles clínicos pediátricos de la migraña vertiginosa tras la enfermedad de Covid-19.	Casos prácticos	El primer caso se refería a un niño que presentaba tres o cuatro veces por semana ataques de vértigo y náuseas, 3 meses después de la infección por el SRAS-CoV-2 y sin otros antecedentes médicos asociados. El segundo caso era un adolescente con quejas diarias de cefaleas e inestabilidad postural, 6 meses después de la infección por SRAS-CoV-2 y sin otros antecedentes médicos asociados. Las intervenciones médicas propuestas para el caso número 1 fueron asumir un estilo de vida equilibrado y una práctica deportiva asociada a la prescripción de refuerzos vitamínicos. En la reevaluación, su estado clínico mejoró. Las intervenciones médicas propuestas para el caso número 2 incluían un estilo de vida equilibrado, pero se aplicó una prescripción farmacológica de un comprimido diario de flunarizina 5 mg.	Migraña vestibular.
Seery et al., 2023, Argentina^(27)^.	Analizar las manifestaciones clínicas de larga duración y las variables de riesgo (margen de unos noventa días) provocadas por la enfermedad causada por el SARS-CoV-2.	Estudio de caso de control	Se analizaron un total de 639 casos de Covid-19, de los que 219 cuidadores declararon síntomas de CL en niños y una edad media de 7 años. La prolongación de los síntomas durante más de tres meses se relacionó con la presencia de enfermedades y disfunciones subyacentes como diabetes, enfermedades renales y de las vías respiratorias, así como con la infección por SARS-CoV-2 sintomática y en un grupo de edad más elevado. No se analizaron los datos de vacunación.	Pérdida de peso, mialgia, agotamiento, taquicardia, dificultad respiratoria, tos, estreñimiento o diarrea, emesis o náuseas, convulsiones e inapetencia, déficit de concentración y memoria, disgeusia, anosmia, vértigo, cefalea, síntomas depresivos y de ansiedad y cambios de humor.
Calcaterra et al., 2024, Italia^(28)^.	Realizar un seguimiento de la historia clínica de los pacientes en edad infantil que hayan recibido atención hospitalaria en por infección aguda por SRAG-CoV-2 para verificar el desarrollo de Covid largo.	Estudio de cohortes	Participaron en el estudio 167 pacientes: el 55% eran menores de dos años, el 30% tenían entre 2 y 10 años y el 14,3% más de 10 años. El 46,1% eran mujeres y el 53,9% hombres. Sin embargo, los autores concluyen que no existe necesariamente una mayor prevalencia en un sexo determinado. Las comorbilidades más frecuentes eran afecciones neurológicas, enfermedades gastrointestinales, afecciones genéticas, diabetes, enfermedades renales, malformaciones cardiacas, obesidad, desnutrición crónica, asma, enfermedades autoinmunes, tumores y otras. No se analizaron los datos de vacunación.	Pérdida de peso, insomnio, sibilancias, gastralgia, fatiga, tos persistente, falta de apetito, mialgia, cefaleas, artralgia y cambios de humor.
Meiere et al., 2024, Letonia^(29)^.	Analizar los grupos de edad infantil diagnosticados de Covid largo al menos 30 días después de la infección primaria por SRAG-CoV-2.	Estudio transversal	La muestra estaba formada por 220 participantes menores de 18 años, 56,8% mujeres y 43,2% hombres. No se analizaron los datos de vacunación ni las comorbilidades.	Epigastralgia, cansancio extremo, cefalea, cambios de humor, limitación de la capacidad física, somnolencia, tos productiva, rinorrea, déficit de memoria, taquicardia y angina de pecho.
Razzaghi et al., 2024, Estados Unidos^(30)^.	Medir los resultados de la protección vacunal contra el desarrollo del síndrome Covid-19 post-agudo en niños y adolescentes (de 5 a 17 años).	Estudio de la cohorte	Se analizaron 480.298 niños de 5 a 11 años y 557.638 preadolescentes y adolescentes de 12 a 17 años. De esta muestra, el 88% de los participantes fueron inmunizados con al menos dos dosis y el 67% con al menos una dosis de la vacuna. La eficacia de la vacuna para proteger contra los síntomas probables de CL fue del 48,2% entre los niños y del 56,1% entre los adolescentes. No se analizaron de forma aislada el sexo y las comorbilidades de los participantes con CL. Entre los participantes que habían estado completamente inmunizados hasta 12 meses, la tasa de protección alcanzó el 45% frente a casos probables de CL. Sin embargo, el factor de protección disminuyó con el tiempo al comparar las tasas de protección contra CL entre seis y doce meses después de la inmunización.	Fatiga, síntomas gastrointestinales, síntomas psicológicos (confusión mental), dolor sistémico persistente.

La revisión mostró que los síntomas de CL notificados con más frecuencia en niños y adolescentes fueron fatiga (10 estudios), cefalea (9), tos (9) y cambios de comportamiento como síntomas psicológicos, trastornos del sueño (8), cambios de humor, ansiedad/depresión (7) y dificultad para concentrarse (7). Otros síntomas frecuentes fueron disnea (6), astenia (3), fiebre (3), pérdida de peso (5), inapetencia (2), disgeusia (5), disosmia (5), dolor abdominal y síntomas gastrointestinales (8), como diarrea (4), náuseas (4), vómitos (3) y estreñimiento (1)^(9,15,17,18,19,20,21,23,24,25,27,28,29)^.

Cabe destacar que los niños de 0 a 4 años suelen presentar una mayor frecuencia de secreciones nasales y tos, síntomas que afectan a los órganos respiratorios^(22,28)^. Por otra parte, los niños a partir de 5 años y, sobre todo, los adolescentes de 11 a 18 años presentan más casos de inestabilidad emocional, menor concentración, trastornos del sueño, ansiedad y depresión, fatiga, cefaleas, disnea y dolores musculares^(9,15,17,20,25,27)^. Además, cuatro estudios han puesto de manifiesto un aumento de la frecuencia de los síntomas de CL a medida que aumenta la edad^(9,19,20,24)^.

Los análisis realizados comparando los sexos de los pacientes fueron divergentes entre los estudios, por lo que resultaron indeterminados, ya que tres estudios mostraron un predominio del diagnóstico de CL entre los niños de sexo femenino^(15,17,21),^ mientras que un análisis señalaba un predominio en los varones^(9)^. Además, un estudio no halló diferencias significativas entre las manifestaciones clínicas de CL en ambos sexos^(28)^.

Sin embargo, ninguno de los estudios analizó la relación entre CL en niños y adolescentes y el género.

En este contexto, solo uno de los estudios evaluó la protección de la vacuna COVID-19 contra CL y demostró que existe una protección temporal contra la probabilidad de desarrollar la afección^(30)^. Cuatro de los estudios también analizaron una mayor frecuencia de comorbilidades entre los pacientes infantiles y adolescentes con CL, como obesidad, enfermedades respiratorias, neurológicas (como epilepsia) y renales^(15,19,20,27)^. Sin embargo, ninguno de los estudios evaluó la asociación entre las comorbilidades y el desarrollo de CL.

## DISCUSIÓN

Se observó una diversidad de síntomas de CL en niños y adolescentes. En este contexto, los síntomas físicos y psicosociales estaban presentes y se manifestaban de forma diferente entre niños y adolescentes. Además, los estudios diferían en cuanto a la incidencia de CL en función del sexo, por lo que los resultados no eran concluyentes, y los diagnósticos secundarios, como la obesidad, resultaron ser importantes en la susceptibilidad a desarrollar CL. Cabe destacar que sólo un estudio analizó la vacunación contra el Covid-19, señalándola como factor protector.

LCL es una afección polifacética que afecta a numerosos sistemas del cuerpo humano, empleando diversos mecanismos fisiopatológicos. El agotamiento prolongado es uno de los síntomas más frecuentes de CL, y su perpetuación puede estar posiblemente asociada a la toxicidad en los tejidos cerebrales debida a la acumulación de líquido cefalorraquídeo provocada por el drenaje linfático en estos órganos, así como a cambios en la función mitocondrial de los músculos y a una disminución del metabolismo cerebral. Además, la inflamación de los tejidos y las lesiones estructurales, especialmente si están causadas por la insuficiencia de oxígeno y de vascularización/microvascularización durante la fase aguda de Covid-19, están relacionadas con daños en el sistema cardiaco^(31)^.

La inflamación persistente y las micro lesiones mencionadas no son exclusivas del sistema cardíaco, sino que también se observan en los sistemas respiratorio, musculoesquelético e integumentario, y pueden conducir al desarrollo de enfermedades autoinmunes. En el sistema inmunitario, el panorama es similar: fomentadas por la inflamación crónica y viendo alterado su equilibrio, las células de este sistema pueden sufrir alteraciones funcionales, como la activación de mastocitos y la activación prolongada de células T^(31)^.

Las lesiones adquiridas en la fase aguda asociadas a este proceso de inflamación crónica pueden provocar a largo plazo daños funcionales en los órganos y estrés oxidativo, potenciado por daños en el sistema cardiocirculatorio. Además, se están investigando otros mecanismos dañinos en los riñones y el sistema gastrointestinal^(31)^.

Teniendo en cuenta este contexto, los resultados obtenidos en esta revisión no fueron concluyentes en cuanto a la manifestación de síntomas de CL analizados en función del género, debido a la divergencia entre los estudios. Al mismo tiempo, en la población general, existe un mayor riesgo de desarrollar CL en las mujeres^(3)^. La mayor prevalencia del diagnóstico de CL en las mujeres puede atribuirse al hecho de que suelen acudir más a menudo a los servicios sanitarios para recibir atención sanitaria rutinaria. Además, otra explicación puede residir en las diferencias inmunológicas basadas en el sexo, que también contribuyen a las variaciones en la incidencia de las enfermedades autoinmunes, que son más frecuentes en las mujeres. Estos factores explican probablemente la mayor prevalencia de CL en mujeres adultas, lo que refuerza la hipótesis de que las hormonas sexuales y su actividad inmuno-moduladora pueden desempeñar un papel importante en el desarrollo de CL en adultos^(32)^.

Del mismo modo, había diferencias en la manifestación de los síntomas de CL por grupos de edad durante CL. Los niños de 0 a 4 años con CL, por ejemplo, presentaban más quejas respiratorias, mientras que los mayores de 5 años tenían más quejas psicológicas. El cuadro clínico físico tanto de la enfermedad aguda como de CL en los niños más pequeños tiende a ser menos expresivo y puede explicarse por mecanismos fisiológicos como el órgano timo operativo en niños de hasta 12 años aproximadamente, así como la importante deficiencia de receptores para el catalizador biológico que convierte la angiotensina II (ACE2), el estado de vacunación y un sistema inmunitario innato significativamente responsivo^(17,28,33,34)^.

Así, en relación con los síntomas físicos, los niveles más elevados de ACE2 y su localización estratégica, la implicación y la exposición a los coronavirus estacionales y a otros agentes víricos, junto con la reactividad cruzada de los agentes inmunizantes, pueden conducir a una restricción de la respuesta inflamatoria a través de la contra-señalización. De este modo, pueden evitarse los síntomas de CL, aunque el organismo se vuelva más susceptible a la propia infección^(33,34)^.

Por otra parte, el umbral de edad para el diagnóstico de CL se define de forma diferente en los distintos estudios seleccionados. Por ejemplo, uno de los estudios muestra una mayor incidencia de CL entre los adolescentes a una edad media de 13 años y otro estudio a los 6 años^(15,20)^. Por lo tanto, puede deducirse que las diferencias en la prevalencia de la clamidia en niños y adolescentes varían según el estudio, probablemente debido a factores que pueden estar relacionados con la gravedad de la infección inicial, así como con sus antecedentes de enfermedades preexistentes^(1)^.

Las manifestaciones sintomatológicas de CL fueron extensas, variadas y expresivas. Así, el aislamiento físico, así como otras medidas sanitarias necesarias para contener la emergencia sanitaria global provocada por la pandemia, pueden haber repercutido en las condiciones clínicas de los niños y jóvenes, favoreciendo la aparición de síntomas como ansiedad, depresión, cambios de humor y trastornos del sueño^(15,22,24,28)^. Hay que tener en cuenta que muchos síntomas físicos, psicológicos y conductuales no son exclusivos de CL, por lo que es difícil para los profesionales sanitarios asociarlos a afecciones aparecidas tras la infección^(1)^.

Bajo el objetivo de mapear los síntomas y el perfil epidemiológico, se encontró que la presencia de comorbilidades y condiciones de salud, factores biológicos y sintomatología de la infección por Covid-19 en la fase aguda pueden interferir con el resultado clínico del desarrollo de CL y aumentar hasta tres veces el riesgo de diagnóstico. Algunos ejemplos son la obesidad, la epilepsia, las enfermedades respiratorias, neurofisiológicas y renales y el dolor muscular durante la infección aguda^(1,35-37)^.

La obesidad fue una de las enfermedades más citadas en los estudios seleccionados. Se observa que la alteración del sistema inmunitario y el diagnóstico de obesidad favorecen la instauración de síntomas de CL debido a los daños causados por la propia infección aguda. Durante la fase aguda de Covid-19, hay una mayor presencia de mecanismos fisiopatológicos en los pacientes con diagnósticos secundarios, especialmente una reacción inflamatoria exacerbada. La obesidad y la infección por SARS-CoV-2 estimulan un entorno inflamatorio importante, y en la obesidad hay una emisión de factores inflamatorios, principalmente el factor de necrosis tumoral alfa (TNFa) (relacionado con la resistencia a la insulina y la diabetes) y las interleucinas 1 y 6 por el tejido adiposo. En la infección por SARS-CoV-2, el estado inflamatorio se asocia a niveles elevados de citocinas, especialmente interleucinas 2 y 7, MCP1, TNFa, factor estimulante de colonias de granulocitos (G-CSF) y la quimioquina CXCL10^(38, 39)^.

En este contexto, el SRAS-CoV-2 estimula una pluralidad de acontecimientos inflamatorios y proinflamatorios que afectan a los sistemas respiratorio, neurológico (células de la microglía, astrocitos, tronco encefálico) y cardiovascular. De este modo, el organismo se vuelve hiperreactivo a la secreción de citoquinas y quimiocinas estimuladas por el virus, favoreciendo los síntomas a largo plazo. Hoy en día, la fatiga, el síntoma más recurrente en los diagnósticos de CL, también puede estar influida por la inflamación crónica del cerebro. Se hipotetizan otros procesos aliados, como el deterioro neuromuscular y las afecciones psicológicas derivadas de la pandemia^(40,41)^.

Al analizar la relación entre el estado de vacunación y CL, cabe destacar que la vacuna Covid-19, además de reducir las probabilidades de desarrollar la enfermedad aguda en su forma grave, también favorece la protección tardía al reducir la presencia de síntomas persistentes. Por lo tanto, no sólo se puede suprimir la probabilidad de infección, sino también su complejidad^(30)^.

Esta información corrobora un estudio brasileño que mostró una mayor incidencia de CL en personas no vacunadas (72%) que vacunadas (59%), lo que pone de relieve la importancia de las estrategias de inmunización y del mantenimiento del calendario de vacunación^(42)^. En Brasil, las vacunas contra el Covid-19 forman parte del esquema de vacunación pediátrica, que consiste en dos aplicaciones, a los seis y siete meses de edad, con cuatro semanas de diferencia. Si el niño tiene el sistema inmunológico comprometido, el programa se realiza en tres dosis y, si no se ha iniciado a la edad preestablecida, puede introducirse hasta los cinco años^(43)^.

Además, el proceso de diagnóstico de CL implica un razonamiento clínico complejo debido a la falta de una prueba específica que identifique con precisión marcadores biológicos relacionados con la enfermedad^(40)^. Se destaca la importancia de los informes de los cuidadores y familiares en la identificación de CL, especialmente en los niños, además de la confirmación de la infección COVID-19 previa mediante una prueba diagnóstica asociada a la exploración física, los análisis de sangre, la función pulmonar, entre otros^(9,15,17,20,23,27,29,44)^.

Así, una sintomatología extensa puede poner en riesgo el bienestar físico y psicosocial de niños y adolescentes, además de deteriorar su calidad de vida y sus actividades cotidianas en una etapa fundamental del desarrollo. Por último, es fundamental realizar un seguimiento continuo de los síntomas, así como llevar a cabo estudios que contribuyan directamente a la práctica clínica, para favorecer la identificación de casos y tratamientos de CL en niños y adolescentes. También es importante profundizar en las condiciones clínicas y enfermedades previas que pueden predisponer al diagnóstico de CL, buscando entender los procesos biológicos en común y cómo pueden agravar la condición clínica en niños y adolescentes.

Las limitaciones de esta revisión son la selección de estudios en portugués, inglés y español, la inclusión de artículos disponibles en su totalidad y la exclusión de bases de datos de indexación no incluidas en la investigación. Sin embargo, este estudio contribuye a identificar los principales síntomas de CL en niños y adolescentes. Con datos específicos sobre CL en niños y adolescentes, será posible desarrollar estrategias de salud pública más eficaces centradas en la prevención, fomentando la vacunación contra Covid-19, el diagnóstico precoz y el tratamiento adecuado. Así pues, conocer los casos de CL en niños es esencial no sólo para proporcionarles un tratamiento adecuado, sino también para mejorar su calidad de vida.

## CONCLUSIÓN

Este estudio mostró variabilidad en los síntomas de CL en niños y adolescentes, con una mayor frecuencia de secreción nasal y tos en los niños de 0 a 4 años, mientras que en los adolescentes de 11 a 18 años hubo una mayor incidencia de inestabilidad emocional, disminución de la concentración, alteraciones del sueño, ansiedad y depresión, fatiga, cefaleas, disnea y dolor muscular. El análisis por sexo no fue concluyente debido a la divergencia de los resultados encontrados en los artículos seleccionados. La vacunación contra el Covid-19 parece ser un factor de protección contra CL.

Por lo tanto, la vacunación es la medida preventiva más eficaz contra CL en niños y adolescentes, lo que refuerza la necesidad de fomentarla en la práctica clínica y priorizarla en las políticas de salud pública en todo el mundo y en Brasil.

Además, a pesar de la creciente atención prestada a la LD en niños y adolescentes, este estudio ha identificado lagunas que requieren una investigación longitudinal más profunda sobre los síntomas de la LD, especialmente estudios estratificados por sexo y grupo de edad. También es importante investigar la correlación entre las comorbilidades y las condiciones socioeconómicas y ambientales y las consecuencias para el desarrollo de CL.

## DISPONIBILIDAD DE DATOS

El conjunto completo de datos que respaldan los resultados de este estudio se publicó en el propio artículo.
